# Light-intensity-dependent photoresponse time of organic photodetectors and its molecular origin

**DOI:** 10.1038/s41467-022-31367-4

**Published:** 2022-06-29

**Authors:** Chiara Labanti, Jiaying Wu, Jisoo Shin, Saurav Limbu, Sungyoung Yun, Feifei Fang, Song Yi Park, Chul-Joon Heo, Younhee Lim, Taejin Choi, Hyeong-Ju Kim, Hyerim Hong, Byoungki Choi, Kyung-Bae Park, James R. Durrant, Ji-Seon Kim

**Affiliations:** 1 Department of Physics, Imperial College London, London, SW7 2AZ UK; 2 Centre for Processable Electronics, Imperial College London, London, SW7 2AZ UK; 3 Department of Chemistry, Imperial College London, London, W12 0BZ UK; 4 Organic Materials Lab, Samsung Advanced Institute of Technology, Samsung Electronics Co. Ltd., Samsung-ro, Yeongtong-gu, Suwon-si, Gyeonggi-do 16678 Korea; 5 Present Address: Advanced Materials Thrust, Function Hub, The Hong Kong University of Science and Technology, Nansha, Guangzhou, Guangdong China; 6 Present Address: Department of Chemical and Biological Engineering, The Hong Kong University of Science and Technology, Hong Kong SAR, China

**Keywords:** Electronic devices, Electronic and spintronic devices, Organic molecules in materials science

## Abstract

Organic photodetectors (OPDs) exhibit superior spectral responses but slower photoresponse times compared to inorganic counterparts. Herein, we study the light-intensity-dependent OPD photoresponse time with two small-molecule donors (planar MPTA or twisted NP-SA) co-evaporated with C_60_ acceptors. MPTA:C_60_ exhibits the fastest response time at high-light intensities (>0.5 mW/cm^2^), attributed to its planar structure favoring strong intermolecular interactions. However, this blend exhibits the slowest response at low-light intensities, which is correlated with biphasic photocurrent transients indicative of the presence of a low density of deep trap states. Optical, structural, and energetical analyses indicate that MPTA molecular packing is strongly disrupted by C_60_, resulting in a larger (370 meV) HOMO level shift. This results in greater energetic inhomogeneity including possible MPTA-C_60_ adduct formation, leading to deep trap states which limit the low-light photoresponse time. This work provides important insights into the small molecule design rules critical for low charge-trapping and high-speed OPD applications.

## Abstract (web summary)

Understanding the charge trapping mechanism in organic semiconductors is crucial to design molecules for high-performance organic photodetectors. Labanti et al. systematically investigate the impact of donor molecular structure on the energetic disorder that affects both shallow and deep trap formation.

## Introduction

Organic semiconductors such as small molecules and conjugated polymers are a new class of attractive photoactive materials for photo-sensing in various imaging applications^1–8^. Their narrow optical absorption bands^9^, combined with highly-flexible, light-weight, transparent, and easily processable physical properties are particularly advantageous for top-surface photodetection in integrated sensor arrays to achieve compact and sensitive high-resolution imaging systems^10,11^. A photoactive layer in particular based on small molecules is usually fabricated as a thin film by a sublimation technique, yielding high batch-to-batch reproducibility^12^. For organic photodiode (OPD) applications, this photoactive layer is typically comprised of a bulk heterojunction (BHJ), a highly intermixed blend of electron donor (D) and electron acceptor (A) molecules fabricated by co-sublimation, which offer efficient separation of excited state excitons into charge carriers at their D/A interfaces^13^. For sublimed OPDs, C_60_ has been the preferential choice as the acceptor molecule due to its isotropic charge transport and ease of sublimation^9–11,14,15^. However, properly selecting and engineering sublimable donor molecules still remain as a key challenge for OPD performance optimization.

Recently, promising OPD performance has been achieved with sublimable donor molecules which possess both donor and acceptor moieties along their conjugated backbone^9,10,15,16^. The resulting push-pull character of these donor molecules provides an effective way to control their electronic energy levels and optical bandgap. Such structural motifs have also enabled, in blends with C_60_, tuning of the photoresponse detection range, enhancement of the charge transport properties, and optimization of the nanostructure of the photoactive layer^9–11,15^. In particular, good performance has been reported with merocyanine and related dyes, achieving a sharp and narrow optical absorption and fast photoresponse^9–11,16^. Various other molecular design strategies such as fusion of the donor moieties and functionalization or elongation of the acceptor moieties have also been explored in the donor molecules, achieving excellent OPD figures of merit including EQEs in the green at 5 V in reverse bias of 60–75%, dark currents of the order of 1 nA/cm^2^, detectivity in the 10^13^ Jones range and linear detection ranges of seven orders of magnitude^9–11,16^.

Despite these achievements, the major limitation of OPDs compared with crystalline inorganic counterparts remains in their relatively slow photoresponse times^17–19^. The charge carrier mobility of organic semiconductors is normally orders of magnitude lower than that of inorganic crystalline materials^20,21^, attributed to polaron formation and higher energetic disorder resulting in charge trapping^22–25^. This results in slower light-on/off photoresponse times^17^ with limited frequency bandwidths in device operation, which are particularly detrimental for imaging reconstruction when OPDs are used in conjunction with faster inorganic detectors in integrated imaging systems^18^. Moreover, even slower OPD photoresponse times can occur in low-light operating conditions^26^, further limiting their potential applications. Energetic disorder and charge trapping in organic semiconductors^27–32^ can stem from multiple molecular origins such as chemical impurity or the co-existence of different conformations (e.g., isomers)^27,32^. Furthermore, molecular-structure induced intermolecular packing, orientation, and domain formation can also contribute to the energetic disorder and charge trapping. Even for sublimed OPDs, known to exhibit better device performance and reproducibility than solution-processed devices, the molecular origins determining these energetic disorder and charge trapping, and hence the OPD photoresponse times, remain largely unresolved^10,11,15,16^.

**Fig. 1:**
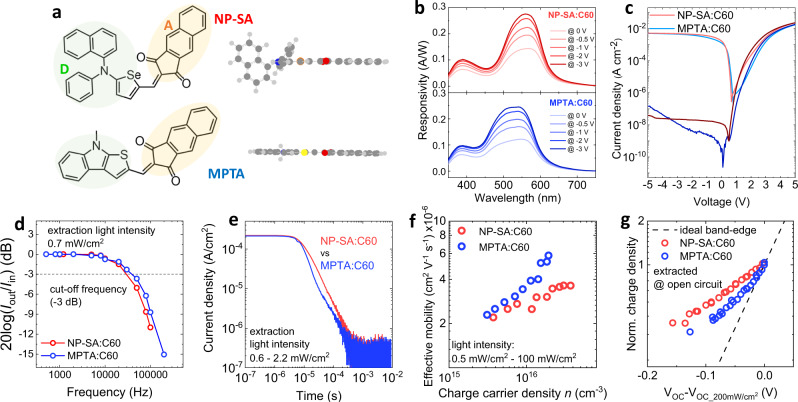
OPD device characteristics at high-light intensity. **a** Molecular structure of NP-SA and MPTA donors, with their donor and acceptor units indicated. The spatial conformation of donor molecules is illustrated based on the DFT optimized geometries shown as side views. **b** The responsivity at different bias conditions. **c** Dark and AM1.5 G light *J*–*V* curves in a semi-log plot, shown with darker and brighter colors respectively. **d** The bandwidth of frequency response measured at a light intensity of 0.7 mW/cm^2^ at 0 V. **e** Light-off photocurrent transient at the light intensity range of 0.6–2.2 mW/cm^2^ illumination at 0 V, the device was illuminated for 100 ms before switching off the light, the background current level is higher than the dark current of the device due to the sensitivity limitation of the detector circuit (50 Ω load resistance, 1 mV/div of the oscilloscope). **f** Effective charge carrier mobilities as a function of charge carrier densities analyzed by charge extraction (CE) at 0 V at the light intensity range of 0.5–100 mW/cm^2^. **g** Normalized plots of the charge carrier density at open circuit versus open circuit voltage determined from integration of charge extraction transients as a function of light irradiation intensity (data normalized at 200 mW/cm^2^ light intensity).

Herein, we elucidate the role of donor molecular structure on the formation of trap states in co-evaporated C_60_ blends, and its impact on the light-intensity dependent photoresponse times of BHJ OPDs fabricated using these blends. Two small molecule donors (NP-SA and MPTA, see Fig. 1 for their chemical structures) are used with C_60_ acceptor in co-sublimed BHJ OPDs. A planar donor MPTA yields a faster OPD response than a twisted donor NP-SA under relatively high-light irradiation (1 Sun down to 0.5 mW/cm^2^). However, OPDs with the planar MPTA donor exhibit biphasic photocurrent transients, with the slower phase dominating at low-light levels. This results in MPTA OPDs exhibiting a slower photoresponse time than NP-SA OPDs when operated under low-light conditions (e.g., 0.02 mW/cm^2^). These light-intensity-dependent photoresponse times are correlated with differences in BHJ nanoaggregation and charge trapping induced by the donor molecular structure (planar or twisted), with in particular the slower photoresponse time of MPTA OPDs at low light levels resulting from a higher density of relatively deep trap states. These relatively deep trap states have negligible impact on high-light OPD operation, but can significantly retard OPD photoresponse time at low-light intensity. In contrast, the more twisted NP-SA donor results in a lower density of these deep trap states, allowing OPD devices to maintain their photoresponse time independent of light intensity. This work provides important insights into the molecular origin of trap states in BHJ OPDs and how molecular design requirements can differ for high- and low-light device operation.

## Results

### Twisted (NP-SA) and Planar (MPTA) donor molecules

The two small molecule donors, NP-SA (2-((5-(naphthalen-1-yl(phenyl)amino)selenophen-2-yl)methylene)−1H-cyclopenta[b] naphthalene-1,3(2H)-dione) and MPTA (2-((8-methyl-8H-thieno[2,3-b]indol-2-yl)methylene)−1H-cyclopenta[b]naphthalene-1,3(2H)-dione), are used (see their chemical structures in Fig. 1a). The synthetic route of NP-SA is reported in ref. ^10^, while full details about MPTA synthesis are included in the Materials section of the Supplementary Methods. Both donors are comprised of electron-donating and electron-withdrawing units (D-A structure) based on fused heterocycle groups, yielding a compact structure suitable for the formation of highly-intermixed interpenetrated networks upon co-evaporation with C_60_^10,11,15,16^. For NP-SA, the donor unit is constituted by a tertiary amine coordinating benzene and naphthalene units to the selenophene molecular core, while three fused rings form its acceptor unit. The optimized geometry of NP-SA based on density functional theory (DFT) shows twisted 3D-like arrangement of donor unit with a planar linkage between the core and the acceptor units (Fig. 1a). For MPTA, the donor unit is made of a single fused heterocyclic group incorporating the amine linkage and the thiophene ring, while maintaining the same three fused ring acceptor unit as NP-SA. This results in an extremely planar molecular structure in MPTA. DFT calculations show the preferential distribution of the highest occupied and lowest unoccupied molecular orbitals (HOMO and LUMO) to the donor and acceptor units respectively for both NP-SA and MPTA molecules (Supplementary Fig. 1). However, the planar MPTA molecule shows smaller electrostatic potential separation on the D and A units, in line with better charge delocalization along its planar conjugated backbone. Although existing studies suggest that the twisted conformation (out-of-plane branching) of the molecule is beneficial to prevent excessive molecular aggregation affecting absorption range and dark current^16^, no detailed study comparing twisted donor molecules to fully-planar ones for BHJ OPD devices has been reported in the literature, which is the main focus of our work.

### Responsivity and current density–voltage characteristics

NP-SA:C_60_ and MPTA:C_60_ BHJ OPDs are green-light selective detectors with a detection range from 460 to 620 nm for NP-SA:C_60_ and 450–600 nm for MPTA:C_60_ (Fig. 1b). Both devices demonstrate a similar level of field-dependent responsivity, assigned to bias-dependent CT dissociation, similar to that previously reported for analogous IDDSe based OPDs^15^. Supplementary Figure 2 shows the photocurrent collection as a function of light intensity at different bias conditions. Non-linear behavior (slope <1) is observed at higher light intensities (*Φ*), indicating bimolecular recombination losses during charge collection, attributed to the highly intermixed nature of the BHJs for both OPDs. Both systems show enhanced linearity at reverse bias, assigned to accelerated charge extraction at reverse bias reducing bimolecular recombination losses during transport. These dependencies are also apparent from the analysis of the derivative of ln*J*/ln*Φ* versus light intensity (Supplementary Fig. 3). Figure 1c shows the *J*–*V* response in dark and under AM1.5 G illumination of both systems. The dark and light *J*–*V* responses are very similar, and the dark currents are particularly low (~10^−8^ A cm^−2^) for a wide reverse bias range of 0 to −5 V when compared with most OPDs^33^. The MPTA:C_60_ device shows a dark shunt increase at strong reverse bias, indicative of a trap associated reverse bias shunt^33^, and consistent with our results below indicating the presence of deep traps in this device.

### High-light device response time governed by shallow traps

To understand the photodetector behavior of NP-SA and MPTA donors, we first consider transient optoelectronic analysis at relatively high illumination intensities (>0.5 mW/cm^2^), employing frequency response analysis, as well as transient photovoltage and charge extraction (CE) techniques more widely applied to organic solar cells^25,27,32^. The frequency response of NP-SA:C_60_ and MPTA:C_60_ OPDs measured at 0.7 mW/cm^2^ (Fig. 1d) exhibits a −3 dB cut-off frequency of 3 kHz for NP-SA:C_60_ and 4 kHz for MPTA:C_60_ (this light intensity was employed to avoid potential contributions from bimolecular recombination losses which will also contribute to the photocurrent response at higher light intensities, see Supplementary Fig. 3). For both devices, the response bandwidth increases at reverse bias −3 V (green and orange data in Supplementary Fig. 4), attributed to accelerated charge extraction due to the larger drift fields in reverse bias conditions (see photocurrent transient data as a function of reverse bias in Supplementary Fig. 5).

The frequency dependent photoresponse of OPDs depends on device circuit RC and charge carrier transport time^17,19^. For low-mobility organic semiconductor-based OPDs, the frequency photoresponse is primarily limited by the charge carrier transport time^17^. Measuring photocurrent transients under pulsed light illumination allows further investigation of the charge carrier transport properties; this method has been applied to study the effective charge carrier mobility of organic solar cells^34^. Figure 1e shows short circuit photocurrent transients of NP-SA:C_60_ and MPTA:C_60_ devices under 0.7 mW/cm^2^. Pulsed light irradiation for 100 ms ensures that the devices reach a steady state precondition with a constant initial light-on current, and the experiments were conducted within the linear dynamic range of the devices (see Supplementary Figs. 2 and Fig. 3). Faster charge extraction transients were observed for MPTA:C_60_ devices at the light intensity ranging from 0.6 to 2.2 mW/cm^2^, consistent with the wider frequency response bandwidth of the MPTA:C_60_ OPDs (Fig. 1d). In particular, a photocurrent decay time of the order of microseconds is in line with state-of-the-art OPD performances^4,15,35^. Integration of the photocurrent transients (see Supplementary Fig. 6) allows the determination of the short circuit charge carrier density at different light levels. These data can then be employed to determine, using a simple drift model, the effective drift charge carrier mobility in the active layer of these devices (see reference ^36^ for calculation details)^36^. For high-light intensities (>0.5 mW/cm^2^), corresponding to charge carrier density higher than ~10^15^ cm^−3^, the effective charge carrier mobility is higher for MPTA:C_60_ compared to NP-SA:C_60_ OPDs (see Fig. 1f).

Charge carrier transport in organic semiconductors is typically dominated by the thermally activated charge hopping processes via band tail states (often referred to as shallow trap states)^37^. For such materials, the mobility is strongly impacted by hopping site density (i.e., trap density) and thermal activation barriers (trap energy)^38,39^. Support for the presence of such shallow trap or tail states in our OPDs was obtained from charge extraction transients measured from open circuit conditions, again as a function of light intensity^34,40^. As can be seen from Fig. 1g, as the open circuit voltage (expected to correspond to the quasi-Fermi level splitting in the photoactive layer) is reduced by lowering the light intensity, the charge density extracted from the devices is lowered (see also Supplementary Fig. 7 for non-normalized data). However, this drop in charge density as a function of *V*_OC_ is significantly shallower than that expected for an ideal semiconductor (see dashed line), evidence for the presence of tail states^27,32^. This drop is steeper for MPTA:C_60_ OPDs, indicative of a narrower energetic distribution of band tail states for the MPTA:C_60_ device compared to NP-SA:C_60_, consistent with the faster response time of MPTA:C_60_ devices under these ‘high-light’ conditions^41^.

### Low-light device response time governed by deep traps

**Fig. 2:**
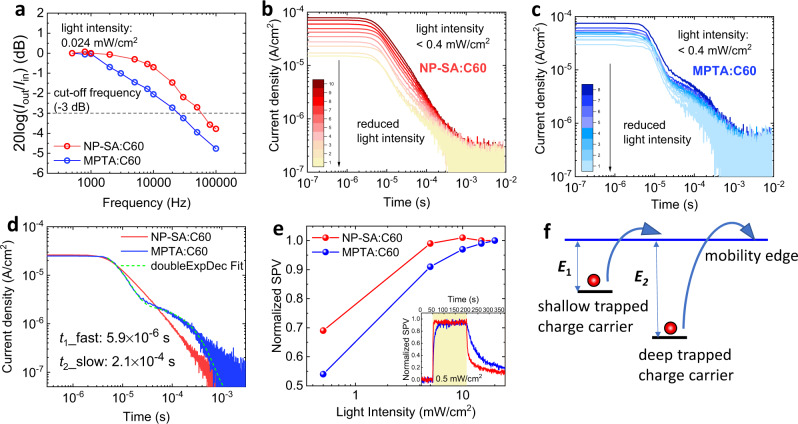
Low-light illumination photocurrent transients. **a** The bandwidth of frequency response at photocurrent density of 2.7 µA/cm^2^ with the light intensity of 0.024 mW/cm^2^ at 0 V. **b** NP-SA:C_60_ device photocurrent transient measured by charge extraction (CE) at 0 V. **c** MPTA:C_60_ device photocurrent transient measured by CE at 0 V. **d** A comparison of current transients measured at 0.5 mW/cm^2^. The photocurrent transient of MPTA:C_60_ is fitted with a double exponential decay function (green dash line). **e** Light intensity dependent SPV magnitude for NP-SA and MPTA blends, normalized to the SPV at the maximum light intensity. The normalized SPV signal during one illumination cycle at the lowest light intensity is shown in the inset. **f** Schematic demonstration of charge extraction of shallow and deep trapped charge carriers.

The operating light intensities of OPDs vary largely between different applications. We therefore now turn to the frequency response and transient extraction analysis of MPTA:C_60_ and NP-SA:C_60_ OPDs at low-light intensities (≤0.5 mW/cm^2^). When the devices are operated at 0.024 mW/cm^2^, the opposite trend in frequency response is observed (Fig. 2a) compared to the high-light case (Fig. 1d). At this low-light intensity, the −3 dB frequency of the NP-SA:C_60_ device is about 5 kHz, while MPTA:C_60_ response time has reduced to 2 kHz. It is thus apparent that MPTA:C_60_ OPD exhibits a superior frequency response for high-light intensity sensing applications, but the NP-SA:C_60_ OPD possesses the fastest response for low-light applications.

To investigate this further, we undertook high sensitivity short circuit photocurrent transients shown in Fig. 2b–d for initial light intensities from 0.1 to 0.4 mW/cm^2^. The initial photocurrent decay kinetics measured at low-light intensity are similar to those observed in Fig. 1e measured at relatively high-light intensities, with faster kinetics for MPTA:C_60_ device. However, as shown in Fig. 2c, it is also apparent that the MPTA:C_60_ device shows a distinct, slower (circa 100 µs) second decay phase which is not significantly observed in the NP-SA:C_60_ devices (Fig. 2b–d). The amplitude of this slow decay phase is observed to saturate at higher-light intensities (see current transients measured at relatively high-light intensities in Supplementary Fig. 8) and is thus more dominant at the lowest light intensities. Therefore, while NP-SA maintains a state-of-the-art photoresponse time^4,15,35^ independently from light intensity, MPTA performance degrades under lower light intensities showing a second slow phase photocurrent transient. Such biphasic photocurrent transients are particularly striking and have not been reported before in previous studies of OPD devices. In particular, the second slow phase decay is evidence of a low density of much slower, but still extractable charge carriers, indicative of the presence of a low density of relatively deep trap states in addition to the band tail states observed near the band edge^42^. Consistent with this observation, light-intensity-dependent surface photovoltage (SPV) measurements also show more prominent deep charge trapping in MPTA:C_60_ OPDs compared to NP-SA:C_60_ devices (Fig. 2e and Supplementary Fig. 9). At this regard, we note that slow charge generation can be ruled out as cause of the observed slow photocurrent transient, since the timescales of the two phenomena are significantly different (i.e., charge generation occurs in the ps-ns range^15^, compared to the 100 µs of the slow charge transient component).

The decay of the MPTA:C_60_ photocurrent transients can be fitted with a biexponential decay function, as shown by the dashed line in Fig. 2d, with a fast decay time constant of 5.9 µs and a slow decay time constant of 210 µs. The difference in these extraction times can be assigned to a difference in effective charge carrier mobility (*µ*) as the extraction speed (*ν*) depends only on mobility when the drift force (*F*) is identical (*ν* = *µF*). Assuming a system with shallow (*E*_1_) and deep (*E*_2_) state energies as illustrated in Fig. 2f, and thermally activated detrapping, the difference in extraction times indicates the deep traps are approximately 0.1 eV below the shallow trap states (we note that this calculation is only indicative, due to its assumption of single trap energies rather than distributions of energies, see Supplementary Note 1 for details). Integration of charge associated with the slow CE decay phase yields the density of these deeply trapped charges, corresponding to 1 × 10^15^ cm^−3^ at short circuit under 0.3 mW/cm^2^. The presence of these deep traps in MPTA:C_60_ devices is also apparent from open circuit charge extraction transients as shown in Supplementary Fig. 7. Open circuit conditions result in enhanced charge accumulation; these data show up to 5 × 10^15^ cm^−3^ charges accumulated in these deep trap states, indicative of the total density of deep trap states in the MPTA:C_60_ OPDs. We note that at short circuit, the charge carrier density accumulating in the device under steady state is lower than at open circuit due to external extraction of photogenerated charge carriers from the photoactive layer^43–45^.

The impact of these deep trap states in MPTA:C_60_ blend can also be probed by light intensity-dependent surface photovoltage measurements (Fig. 2e)^46^. The magnitude of SPV response is significantly dependent on light intensity for MPTA:C_60_ blend, showing a strong reduction compared to NP-SA:C_60_ blend when measured in low-light (notably at 0.5 mW/cm^2^), indicating strong trap-assisted recombination loss, consistent with deep trap states being more dominant in MPTA blends. Moreover, MPTA:C_60_ blend shows a slower transient time to reach equilibrium in the dark after low-light illumination (shown in the inset in Fig. 2e), which suggests that these deep trap states slow down charge extraction in MPTA:C_60_ blend^47,48^. In summary, we conclude that the slower frequency response of MPTA:C_60_ OPDs at low-light intensities results from the presence of relatively deep trap states, while these are not so prevalent in NP-SA:C_60_ OPDs.

### Molecular-structure-dependent intermolecular coupling

**Fig. 3:**
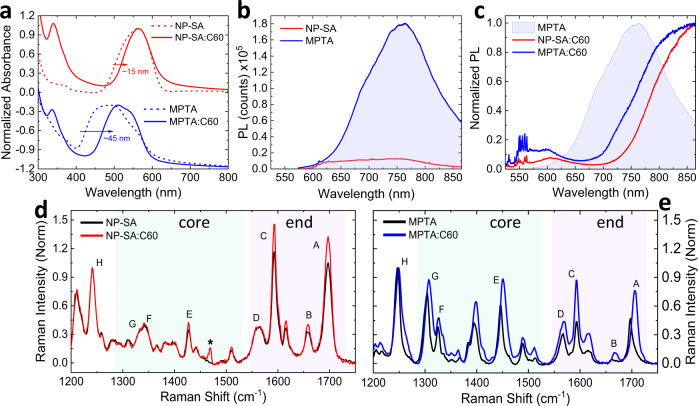
Optical and molecular structural properties. **a** Normalized UV-Vis absorption spectra for NP-SA, MPTA and respective C_60_ blends. **b** Absolute photoluminescence (measured by 514 nm excitation and corrected for absorbance) for neat donor thin films. **c** Normalized PL for blends and neat MPTA, measured by 514 nm excitation. **d**, **e** Comparison between normalized Raman spectra (via 488 nm laser excitation) of neat and blended NP-SA and MPTA films. The peaks are labeled according to the detailed assignment shown in the Supplementary Information and grouped according to core and end-group vibrational modes. The asterisk in (**d**) indicates the C_60_ vibrational mode appearing in blends.

In order to identify possible molecular origins for shallow and deep traps, we first investigate the effect of donor molecular structure (twisted NP-SA vs planar MPTA) on intermolecular coupling in neat and blend films using optical and structural probes. For absorption, both donor molecules show a well-defined absorption band in the visible range with a smaller (90 nm) full-width at half maximum (FWHM) peaking at 565 nm for NP-SA and a larger (150 nm) FWHM peaking at 495 nm for MPTA, respectively (Fig. 3a). For MPTA, a clear absorption feature at the low energy shoulder is also observed. Surprisingly, both NP-SA and MPTA absorption spectra get narrower, in particular for the high energy shoulder, upon blending with C_60_, and this narrowing is more dominant in MPTA, reducing its FWHM by 45 nm compared to 15 nm in NP-SA (Fig. 3a).

More striking differences between the two donors appear in their photoluminescence (PL) spectra (Fig. 3b). The planar MPTA shows strongly red-shifted, highly-emissive PL emission compared to the twisted NP-SA, with its PL intensity increasing further upon thermal annealing (Supplementary Fig. 10), indicating stronger intermolecular interactions of MPTA most likely associated with molecular aggregation, arising from its extremely planar molecular structure. Similar emission characteristics with strongly red-shifted and highly-emissive PL has also been observed in analogous organic planar small molecules, assigned to excimers^49^. The lifetime of these emissive species is found to be in the order of 5 ns by time-resolved PL (Supplementary Fig. 11), which is consistent with excimeric emission reported^50,51^, while NP-SA shows a much faster decay (<400 ps limited by the instrument response function). The aggregation in MPTA seems to occur at the molecular level without any detectable microscopic features (Supplementary Figs. 12 and 13 for atomic force microscopy (AFM) and transmission electron microscopy (TEM) images, respectively). Upon blending with C_60_, this red-shifted, highly emissive PL of MPTA is strongly quenched, while a new broad charge transfer (CT) state emission appears at low energy (>800 nm) (Fig. 3c). This CT state emission originates from weakly bound electron-hole pairs formed upon photoexcitation at the MPTA/C_60_ interfaces; this is readily quenched by reverse electric field generating free charge carriers (Supplementary Fig. 14). A similar red-shifted CT state emission is also observed in NP-SA:C_60_ blend. Such dominant CT state emission in both MPTA:C_60_ and NP-SA:C_60_ blends is indicative of highly intermixed morphology for both blends^15^. Differently from NP-SA:C_60_ showing stronger and predominant CT emission, MPTA:C_60_ blend emission shows more visible high energy shoulder (<750 nm) coinciding with MPTA emission, indicating possible presence of residual neat MPTA emission. For both blends, a new additional blue-shifted, much weaker PL emission is also observed at high energy (<650 nm) with higher relative intensity for MPTA:C_60_ blend, attributed to the emission of donor molecules where their intermolecular interactions are largely disturbed by C_60_ acceptors. Based on these absorption and PL properties, it is clear that the planar donor MPTA undergoes more significant changes in the molecular-scale nanomorphology to accommodate C_60_ acceptor in the blend than NP-SA.

**Fig. 4:**
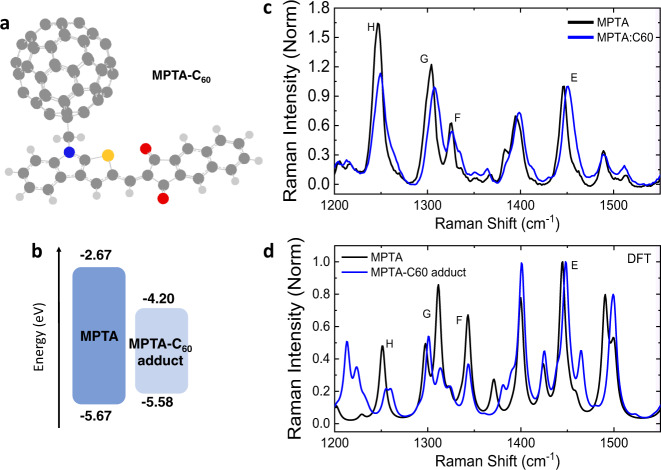
MPTA-C_60_ adduct. **a** Chemical structure of MPTA-C_60_ adduct, in DFT optimized structure. **b** Calculated energy levels for MPTA and MPTA-C_60_ adduct. **c** Measured Raman spectra of neat and blended MPTA, normalized to peak E from the core thiophene. **d** DFT simulated Raman spectra for MPTA and MPTA-C_60_ adduct.

These significant changes in the nanomorphology of MPTA:C_60_ blends are also apparent from molecular vibrational spectroscopy. Figure 3d, e shows the Raman spectra of NP-SA and MPTA respectively, comparing neat and blend films. The main peaks appear at low (1300–1500 cm^−1^) and high (1550–1800 cm^−1^) frequencies, which mainly originate from the core unit (i.e., central selenophene/thiophene and amine linkage) and the end-group (i.e., acceptor unit, phenyl, and naphthalene) vibrations of molecules^15^, respectively (see Supplementary Fig. 15 and Supplementary Table 1 for detailed assignment). In neat films, MPTA shows much stronger and sharper core unit peaks, indicating enhanced π-electron density in the fused donor unit compared to non-fused donor in NP-SA^52–54^. Blending with C_60_ leads to completely different changes in their vibrational modes. For NP-SA, it affects only the high energy vibrations (peaks A and C) with increased relative intensities, consistent with a certain degree of molecular twisting selectively involving only the end-groups^15^. On the other hand, the blending induces significant structural changes in MPTA, with increased relative intensities of all the vibrations accompanied by large broadening of the peaks towards high frequencies. This does not match the Raman spectra of twisted MPTA (see Supplementary Fig. 16), indicating that molecular twisting is not the main cause of Raman changes observed in MPTA:C_60_ blend. Such significant changes reflect the more dramatic impact that C_60_ blending has on MPTA, disturbing its strong intermolecular coupling^52,55–58^. As planar MPTA exhibits strong intermolecular coupling in neat film, as evident in its red-shifted and highly emissive PL, the addition of C_60_ requires profound modifications of MPTA packing and relative molecular orientation for an effective mixing, leading to significant changes in MPTA:C_60_ nanomorphology, as probed herein by Raman spectroscopy. It is striking that these changes of nanomorphology occur at the molecular scale without any measurable changes in microscopic morphology (Supplementary Figs. 12 and 13). In addition to morphology reorganization, the blending of C_60_ can result in the formation of MPTA-C_60_ adducts^59–61^ (see chemical structure and calculated energy levels in Fig. 4a, b), only possible in the planar MPTA where there are no bulky side groups at the amine center and hence the lone electron pair in the N atom can interact with π-electrons in C_60_. Figure 4c, d shows the calculated Raman spectrum of the MPTA-C_60_ adduct, which seems compatible with the experimental data for the MPTA blend. In fact, both show similar changes in Raman intensity and peak width compared to neat MPTA. Namely, the relative quenching of peaks H and G can be attributed to the locking of the amine bond by the fullerene addition, and the broadening of peak E towards high energies can be related to adduct contributions. In summary, we find that the MPTA-C_60_ adduct formation is possible in MPTA:C_60_ blend due to the extremely planar structure of MPTA. When C_60_ is highly intermixed with MPTA in blend, the strong intermolecular interactions of MPTA molecules are disturbed, allowing MPTA-C_60_ interaction to form MPTA-C_60_ adduct through lone pair–π interactions^60,61^. Both morphology changes and adduct formation significantly affect the MPTA vibrational modes, consistently with what observed from Raman measurements.

### Molecular origins of shallow and deep traps

**Fig. 5:**
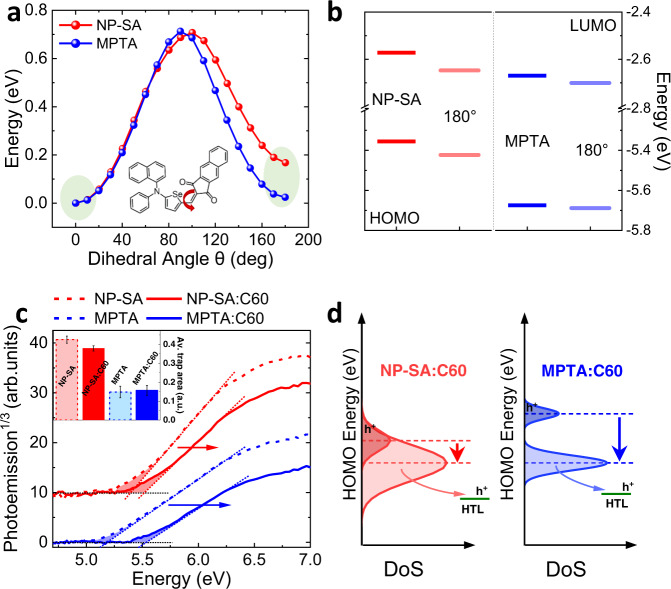
Energetic analysis of NP-SA:C_60_ and MPTA:C_60_ blends. **a** The potential energy scan as a function of alkene bond dihedral angle between donor and acceptor units (e.g., as indicated for NP-SA in the inset), with highlighted the lower energy configuration at 0 and 180° dihedral angles for NP-SA and MPTA. **b** HOMO and LUMO energy levels for optimized geometries of NP-SA and MPTA and their isomeric forms, respectively with 0 and 180° D-A dihedral angle, calculated by DFT. **c** Photoemission spectra of neat and blended donors measured by APS, with the linear fits for the location of emission edge attributed to HOMO (dotted lines). The inset shows the density of tail states extracted from the integrated area below the HOMO onset, indicative of shallow trap density, with error bars as standard deviation across multiple measurements. **d** Distribution of deep and shallow trap states. MPTA:C_60_ has a narrower shallow trap distribution and the presence of a deeper trap DOS. The trap states are filled by photogenerated holes from low energy states to high energy states gradually with the increase of light intensity. In low light, hole extraction from deep states of MPTA:C_60_ is harder than the extraction from shallow states, due to the higher energy barrier, leading to hole trapping in deep states.

We now turn our investigation into the effect of donor molecular structure (twisted NP-SA vs planar MPTA) on the formation of shallow and deep trap states in BHJ blends using DFT simulation and energetics measurements. We first analyze the conformation of the two donor molecules. Figure 5a shows an analysis of molecular potential energy as a function of structural twisting using DFT simulations, in which the potential energy is calculated at different dihedral angles between the D-A building blocks—i.e., between selenophene (or thiophene) and alkene bond in the A unit for NP-SA (or MPTA). Both donor molecules exhibit a planar D-A geometry (0 dihedral angle) at the lowest potential energy, with the chalcogen and oxygen atoms at the same side of the structure, lowering the potential energy through their non-covalent bond. Notice that the optimized NP-SA geometry has a 3D coordination in the amine donor unit, but a planar conformation between the selenophene core and the acceptor unit (Fig. 1a). For NP-SA, a large potential energy difference (~200 meV) is calculated for its isomer, where the dihedral angles between the D-A building blocks is 180°. In contrast, for MPTA the two isomers (0 and 180° dihedral angles) have a very similar potential energy, attributed to the weaker S–O interaction in MPTA compared to stronger Se–O interaction in NP-SA. The HOMO and LUMO energy levels calculated for the two isomers of MPTA are also very similar (Fig. 5b), while they are largely different (~100 meV) for NP-SA. This holds even considering the possible non-equilibrium configurations (20° around the local minima), which can represent the highly intermixed blend morphology. The HOMO level dispersion is ~70 meV for NP-SA, but only ~30 meV for planar MPTA (see Supplementary Fig. 17). This indicates an important intrinsic structural advantage of the planar MPTA molecule. Even with the possible formation of isomers in films, no additional conformational energetic disorder is introduced by this planar molecule, leading to narrow band-tail-state distribution (low density of shallow traps), consistent with the fast charge transport and fast OPD photoresponse time at high-light levels. At this regard, it is important to point out that selenation plays a role in the stabilization of the molecular potential energy minimum, therefore on the propensity for the formation of isomers. However, the changes in DOS distribution upon creation of isomers are mainly dictated by the specific molecular structure of different donors (i.e., planar vs twisted). In fact, as visible in Supplementary Fig. 18, the selenated equivalent of MPTA has a stronger potential difference between optimized and 180° conformations similarly to NP-SA, but the difference in HOMO and LUMO levels between isomers remains low (~30 meV).

In order to gain further insight into the molecular origin of trap states, we measure the energetics of neat and blend films using ambient photoemission spectroscopy (APS) measurements^32^. The measured HOMO of neat MPTA is much shallower than that of NP-SA (5.16 eV vs 5.37 eV) (Fig. 5c). This indicates the strong intermolecular coupling of the planar MPTA in solid state, leading to a ~0.50 eV shallower HOMO compared to single-molecule gas-phase DFT simulations, while measured HOMO is consistent with the calculated value for NP-SA. Upon blending with C_60_, a clear HOMO level deepening is visible for both donors, with a much larger shift for MPTA (370 meV vs 100 meV for NP-SA). This is accompanied by a more prominent narrowing of the density of states (DOS) in MPTA blend (Supplementary Fig. 19). Analysis of sub-gap features from the APS spectra^32^ (see the inset in Fig. 5c) clearly reveals a low density of tail states intrinsic to the planar MPTA, which is preserved even after blending. This low density of tail states near the band edge (i.e., shallow trap states) is consistent with the narrow band-tail state distribution extracted via charge extraction transients (Fig. 1g). We attribute this low density of shallow trap states to the smaller energetic disorder intrinsic to the highly planar MPTA, with its potential isomer formation not introducing additional conformational energetic disorder. This low density of shallow trap states and stronger intermolecular coupling leads to the superior charge transport and fast photoresponse time in MPTA:C_60_ OPDs when operating at higher light levels (Fig. 1e).

The highly planar MPTA exhibits strong intermolecular coupling in neat film forming a low-bandgap, highly-emissive electronic state (see excimeric emission in Fig. 3b). However, the strong disturbance induced by C_60_ in BHJ blend induces significant changes in MPTA, breaking strong intermolecular interactions between MPTA molecules and introducing a new larger-bandgap MPTA electronic state with significantly lowered HOMO level in the blend. As such, any residual low-bandgap electronic states present in the blend will act as intraband deep trap states. Along with the remaining aggregation of neat MPTA, also the formation of a small density of MPTA-C_60_ adducts^59–61^ would result in additional energy levels. According to calculated energetics of MPTA-C_60_ (Fig. 4b), the adduct exhibits a ~90 meV shallower HOMO level compared to MPTA, which can also act as a deep trap state in MPTA:C_60_ devices.

This provides a molecular origin for the slower photoresponse times observed for MPTA:C_60_ devices at low light intensities, with these states resulting in the slow photocurrent decay phase and the strong light intensity dependence of SPV shown in Fig. 2.

## Discussion

In this study, we have elucidated the molecular structure-dependent charge trapping and its impact on OPD light-intensity-dependent photoresponse times, which is crucial for further developing organic molecules for targeted OPD applications. The control in the density of shallow trap states achieved in MPTA by its highly planar molecular conformation is a key factor for the fast charge transport and the fast photoresponse time under standard illumination conditions (high-light intensity) observed in MPTA:C_60_ devices. However, this highly planar molecular structure leads to strong intermolecular coupling between MPTA molecules forming a low-bandgap state. In blends with C_60_, this coupling is largely disrupted, resulting in shift of HOMO level for most MPTA molecules of several hundred meV (see schematic in Fig. 5d). It is likely that residual low-band-gap electronic states and the additional formation of MPTA-C_60_ adducts are the primary origin of deep charge trapping which under low-light excitation conditions leads to the biphasic photocurrent transients and limited photoresponse bandwidth observed for MPTA:C_60_ OPDs. No such deep trap states are expected in NP-SA:C_60_ devices as C_60_ blending has minor impact on NP-SA morphology and energetics due to its twisted and more amorphous structure. Moreover, the stronger steric hindrance by the bulky phenyl and naphthalene groups in NP-SA prevents the amine from reacting for the formation of C_60_ adducts. This more amorphous morphology, with weaker intermolecular interactions, results in a slower photoresponse time under standard operation conditions, making the highly planar MPTA a preferential candidate for high performance OPDs. However under low-light conditions, the performance of MPTA:C_60_ OPDs is impeded by deep charge trapping resulting in NP-SA:C_60_ OPDs yielding faster response times for applications requiring low-light operation.

## Methods

### Fabrication of devices and thin films

All organic semiconductor materials were purified via sublimation under high vacuum (<10^−6^ Torr) prior to use. Thin films were fabricated via thermal evaporation under high vacuum (<10^−7^ Torr) at a rate of 0.35 A/s on dried glass or quartz substrates that had been cleaned with isopropyl alcohol (IPA) and acetone in an ultrasonic bath. The OPDs were fabricated on ITO-coated glass substrates by sequentially depositing the hole-extraction layer (5 nm), the organic BHJ layer of Donor:C_60_ (100 nm, 1:1 w/w), electron extraction layer, Ytterbium (2 nm) and an ITO electrode (10 nm) (Supplementary Fig. 20). All organic film layers were thermally evaporated under high vacuum (<10^−7^ Torr). ITO electrodes were evaporated by magnetron sputter before the devices were finally encapsulated with glass (98.5% transmittance). The active pixel size was 0.04 cm^2^. Thermal annealing post-fabrication of thin films was performed at 160 °C over a hot plate for 3 h, followed by natural cooling to room temperature.

### Transient optoelectronic measurements

The basic set-up of the transient optoelectronic measurements for fully encapsulated devices employed in this work used an oscilloscope (Tektronix TDS 3032B) to record the transient signal. A ring of 12 Luxeon Star/O (LXHL-NWE8) white LEDs was applied to supply light illuminations (see LED spectrum in Supplementary Fig. 21). The bias supply was a Keithley 2400 source meter to provide different voltage conditions to the device. The low-light conditions were applied via LED power control, combined with additional Neutral Density Filters to achieve the lowest levels of light intensity. Low current values were amplified via a low noise amplifier (DLPCA-200 - FEMTO Current Amplifier) to be detected by the oscilloscope. All of these measurements were controlled via computer with in-house software written in Wavemetrics IGOR Pro software.

### Steady-state optical and structural characterization

PL and Raman spectra were measured by a Renishaw inVia Raman microscope in backscattering configuration. The samples were kept under a constant nitrogen flow in a Linkam chamber to reduce degradation effects. Laser excitation wavelengths were 514 nm for PL and 488 nm for Raman measurements (Argon, Titanium Sapphire lasers), with a laser spot diameter of the order of 10 µm, 25% defocused on the sample. Diffracted light was separated by a diffraction grid (2400 lines/mm for Raman spectra and 300 lines/mm for PL). A Si reference sample was used for spectrometer calibration. Optimized acquisition parameters (laser power, exposure time, measurement accumulation number) were kept consistent for the same experiments. Accuracy was improved by checking the reproducibility of spectra over multiple positions on the surface. PL background was removed from Raman spectra by polynomial fitting. PL was also measured on encapsulated devices applying reverse bias to the electrodes. Transmittance (*T*) of thin films was measured by a Shimadzu UV-2550 UV–Vis spectrophotometer, converting it into absorbance (*A*) and removing the substrate contribution by the equation *A* = log(*T*_substrate_/*T*_sample_), with the approximation of no reflectivity.

### Energetic and morphological characterization

Materials HOMO energy levels were measured for thin films on grounded ITO substrates using an APS04 photoemission system (KP Technology) in ambient conditions with a 2 mm gold tip. Reproducibility check and experimental error calculation were carried out by measuring at multiple positions on the surface. APS data were analyzed according to the protocol described by Baikie et al.^62^, extracting the HOMO from the crossing between the linear fit of the photoemission intensity cube root and the zero emission baseline. DOS was extracted as first derivative from photoemission cube root spectra by software analysis. The same system was used as Kelvin probe to measure the changes in surface potential in cycles of dark and illuminated conditions (white bulb light source, with tunable intensity up to 1/5 Sun). Surface morphology was measured by a Park NX10 AFM in tapping mode with Park silicon PPP-NCHR tips combined with SmartScan software.

### Computational methods

Simulations based on DFT were carried out using Gaussian09 software on the Imperial College High Performance Computing service. GaussView 6 was used as a graphical interface for result visualization. DFT was applied at the B3LYP level with 6-311G(d,p) basis set. Small molecule donor molecular structures were optimized to the minimum energy in gas phase and dihedral scans were performed freezing the chosen dihedral coordinate and optimizing the molecular structure at each step. Raman vibrational modes were simulated for peak assignment of experimental data, applying a wavenumber scaling factor of 0.97 for consistency^63^.

## Supplementary information

Supplementary Information

## Data Availability

The data supporting the findings of this study are available from the corresponding author upon reasonable request.

## Code availability

The codes or algorithms used to analyze the data reported in this study are available from the corresponding authors upon reasonable request.
