# Supplementary material for: Light-intensity-dependent photoresponse time of organic photodetectors and its molecular origin
Source: Nat Commun. 2022 Jun 29;13:3745. doi: 10.1038/s41467-022-31367-4 (PMC9243077; doi:10.1038/s41467-022-31367-4)
Supplement: Supplementary file 1 — Supplementary Information [file 41467_2022_31367_MOESM1_ESM.pdf]

Supplementary Information

Light-Intensity Dependent Photoresponse Time of Organic Photodetectors and Its Molecular Origin

Chiara Labanti[#] , Jiaying Wu[#], Jisoo Shin[#] , Saurav Limbu, Sungyoung Yun, Feifei Fang, Song Yi Park, Chul-Joon Heo, Younhee Lim, Taejin Choi, Hyeong-Ju Kim, Hyerim Hong, Byoungki Choi, Kyung-Bae Park*, James R. Durrant* and Ji-Seon Kim*

[#]The first three authors contributed equally to this paper.
*Corresponding author.

Supplementary Figures

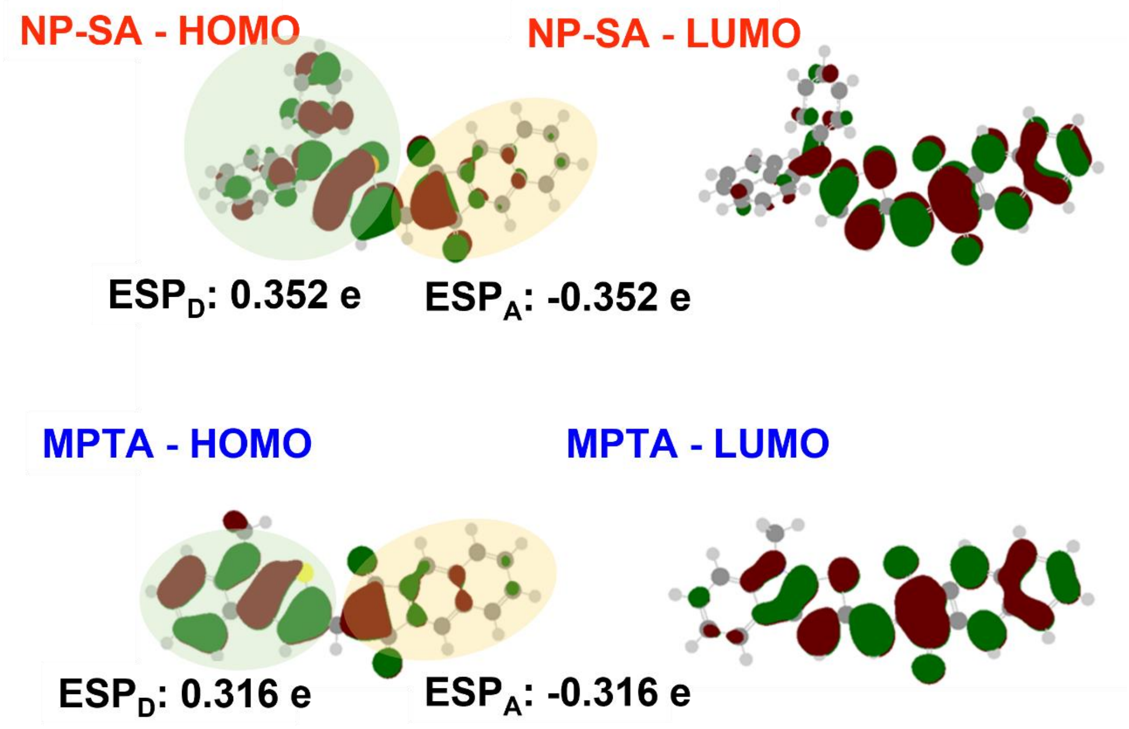

Supplementary Figure 1. Spatial distribution of HOMO and LUMO for NP-SA and MPTA molecules in their optimized geometry calculated by DFT. Simulated electrostatic potentials for donor and acceptor units (ESP_D and ESP_A, calculated based on Merz-Kollman model) indicate a smaller charge separation in the donor/acceptor units for MPTA, according to the more effective charge delocalization in a planar molecular structure.

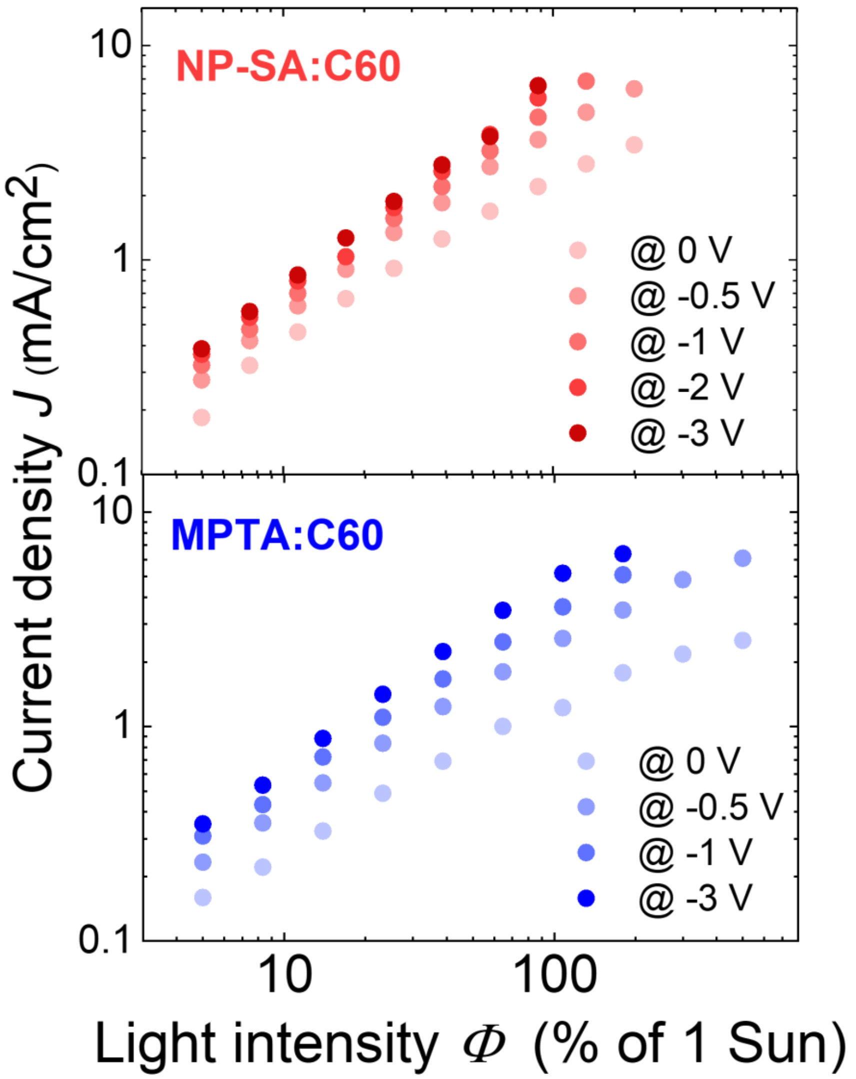

Supplementary Figure 2. Extracted photocurrent as a function of light intensities at different bias conditions for NP-SA and MPTA devices.

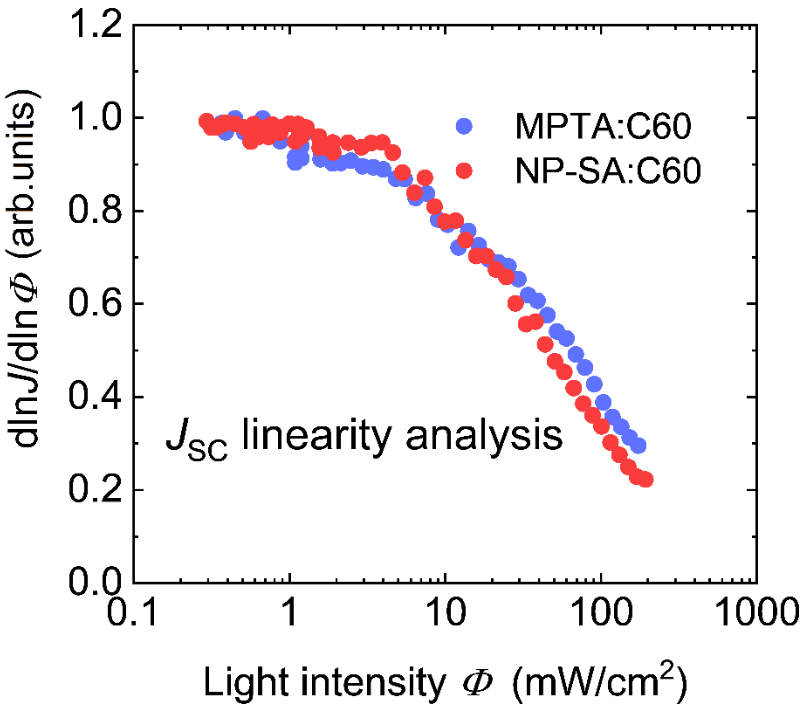

Supplementary Figure 3. Linearity analysis of photocurrent collection in short circuit condition as a function of light intensities of NP-SA:C_{60} and MPTA:C_{60} devices. The drop of linearity below 1 is assigned primarily to bimolecular recombination accelerating at higher light intensities.

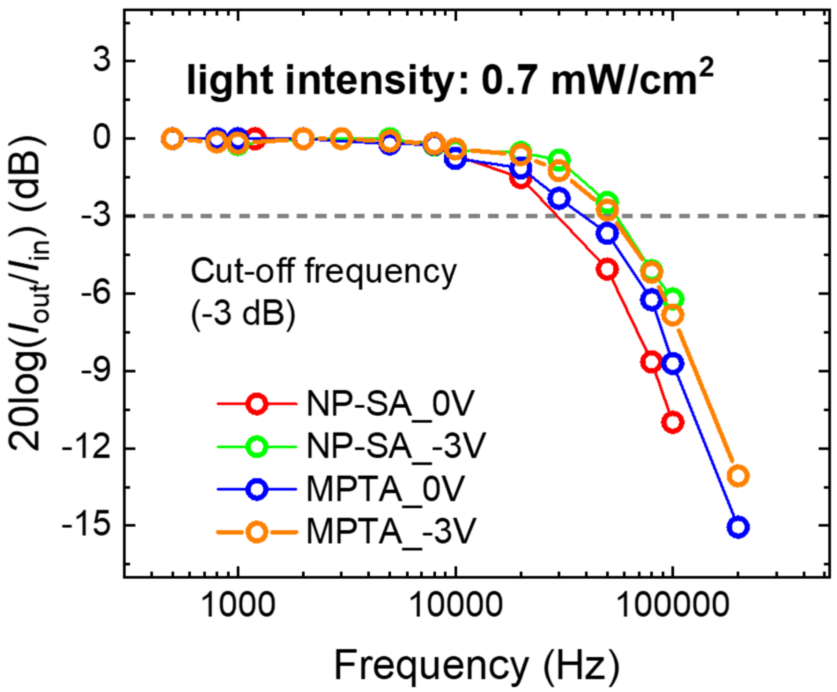

Supplementary Figure 4. Bias dependent frequency response at the light intensity of 0.7 mW/cm^2 at 0 V and -3 V.

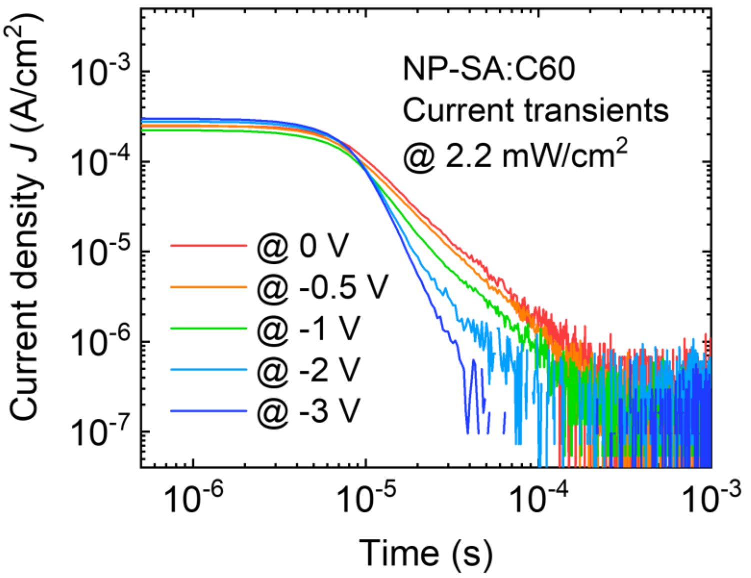

Supplementary Figure 5. Photocurrent transients of NP-SA:C_{60} device at the light intensity of 2.2 mW/cm^2 at different bias from 0 V to -3 V.

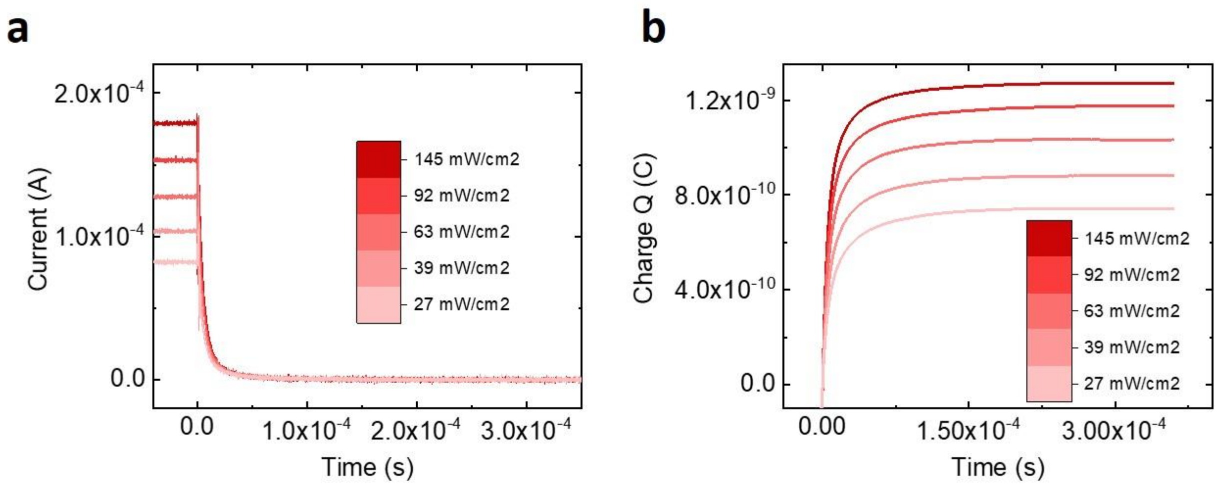

Supplementary Figure 6. (a) Photocurrent transients on as linear-linear scale. (b) The corresponding integration of (a).

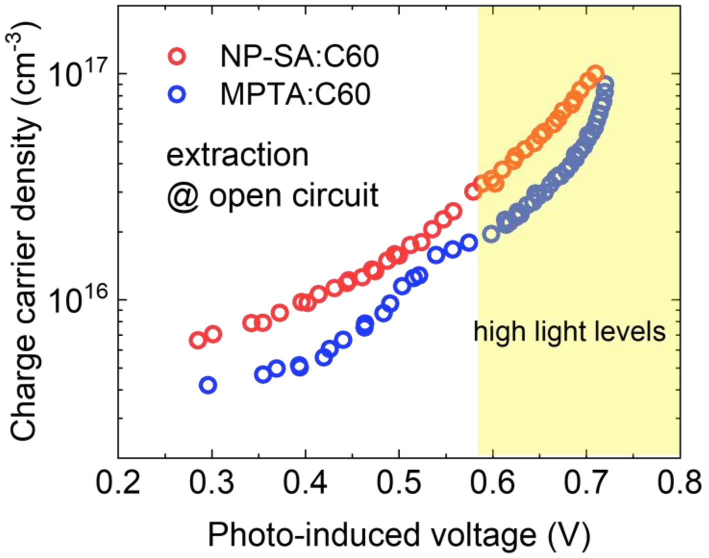

Supplementary Figure 7. Charge extracted at open circuit. The data shown in the yellow area are extracted at high light intensities.

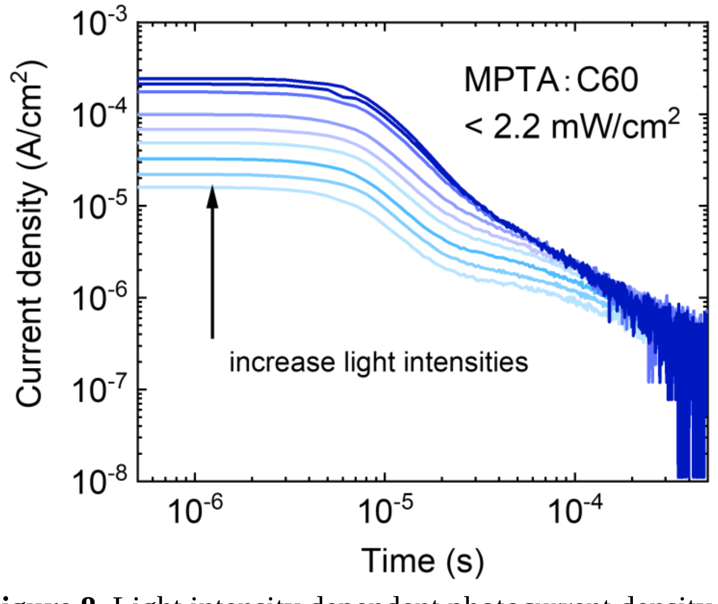

Supplementary Figure 8. Light intensity dependent photocurrent density transients of MPTA:C_{60} measured at 0 V.

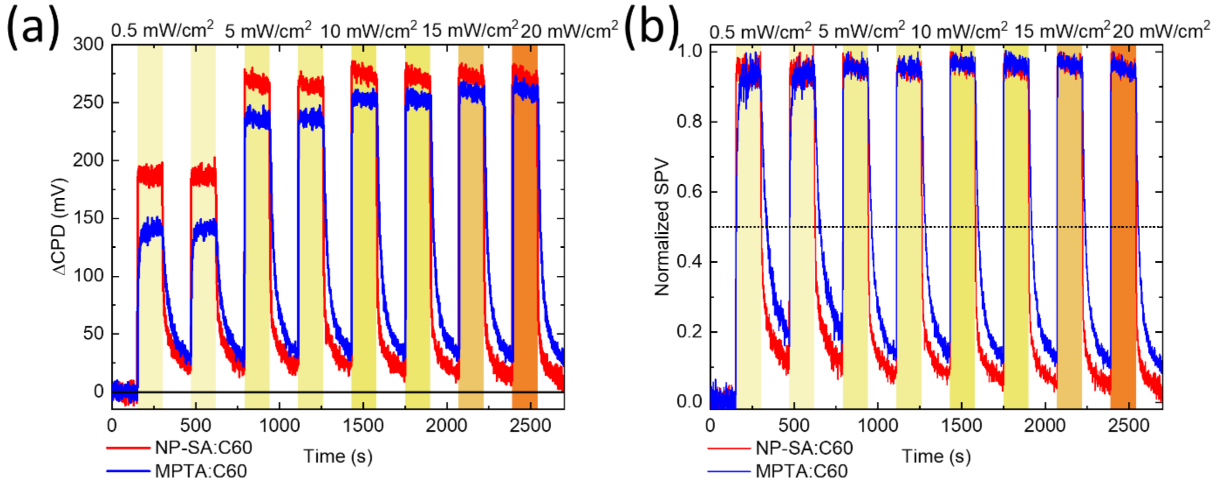

Supplementary Figure 9. Absolute (a) and normalized (b) SPV response for NP-SA and MPTA blends scanning through increasing white light intensities. The active layer is deposited on Ag substrate with hole extraction interlayer in between. *Notes on SPV analysis*: the technique monitors the changes in contact potential difference (CPD) between a metallic tip and the sample surface. The SPV is defined as CPD difference between dark and illuminated conditions. The positive signal observed in light corresponds to electron accumulation on the surface after exciton generation and separation upon light excitation. This is understandable with the film being deposited on hole extraction interlayer (IL) and Ag. The magnitude of SPV signal and the turn-on/turn-off dynamics can be related to transport efficiency, presence of trap states and interfacial energetic offsets.

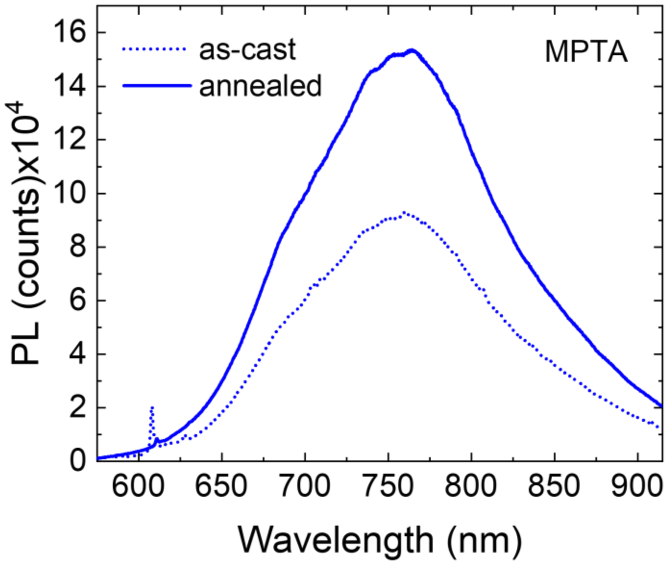

Supplementary Figure 10. Absolute photoluminescence for pristine and annealed MPTA films under 514 nm laser excitation, corrected for absorption at laser wavelength.

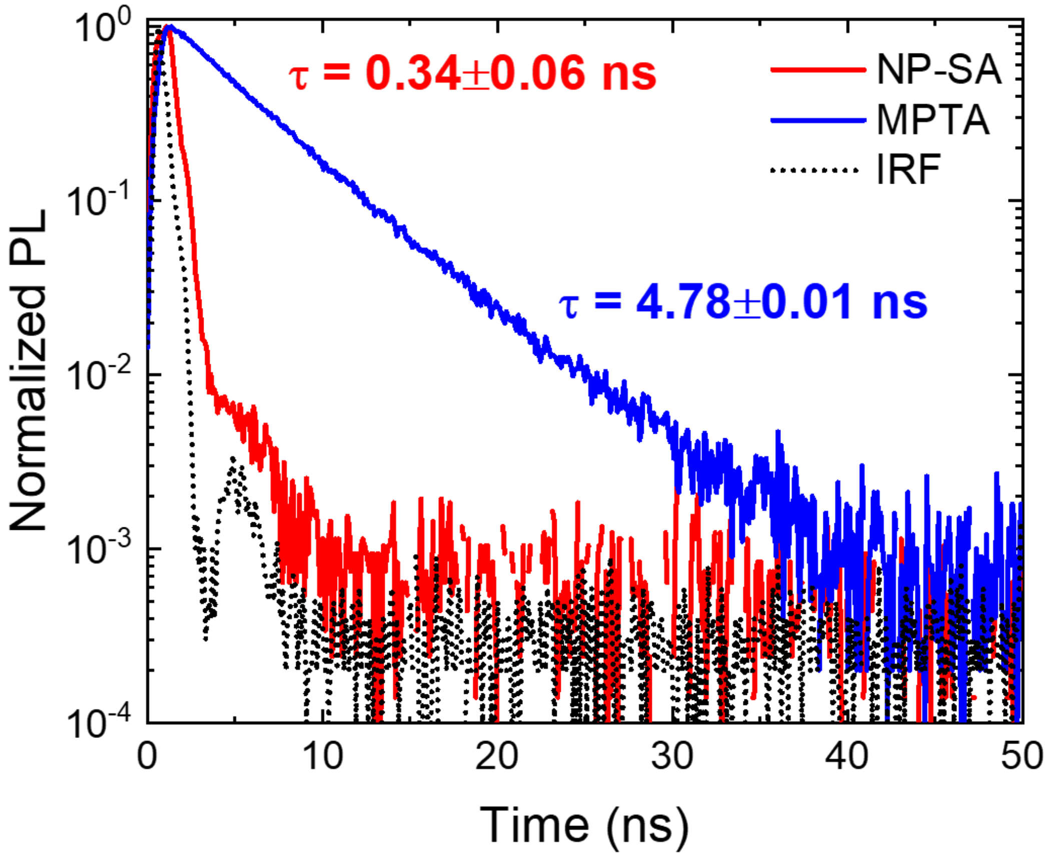

Supplementary Figure 11. Transient PL for NP-SA and MPTA, respectively monitored at 750 and 770 nm following 405 nm laser excitation. The lifetimes from single exponential fit are indicated in the graph. Instrument response function (IRF) is also included as a reference.

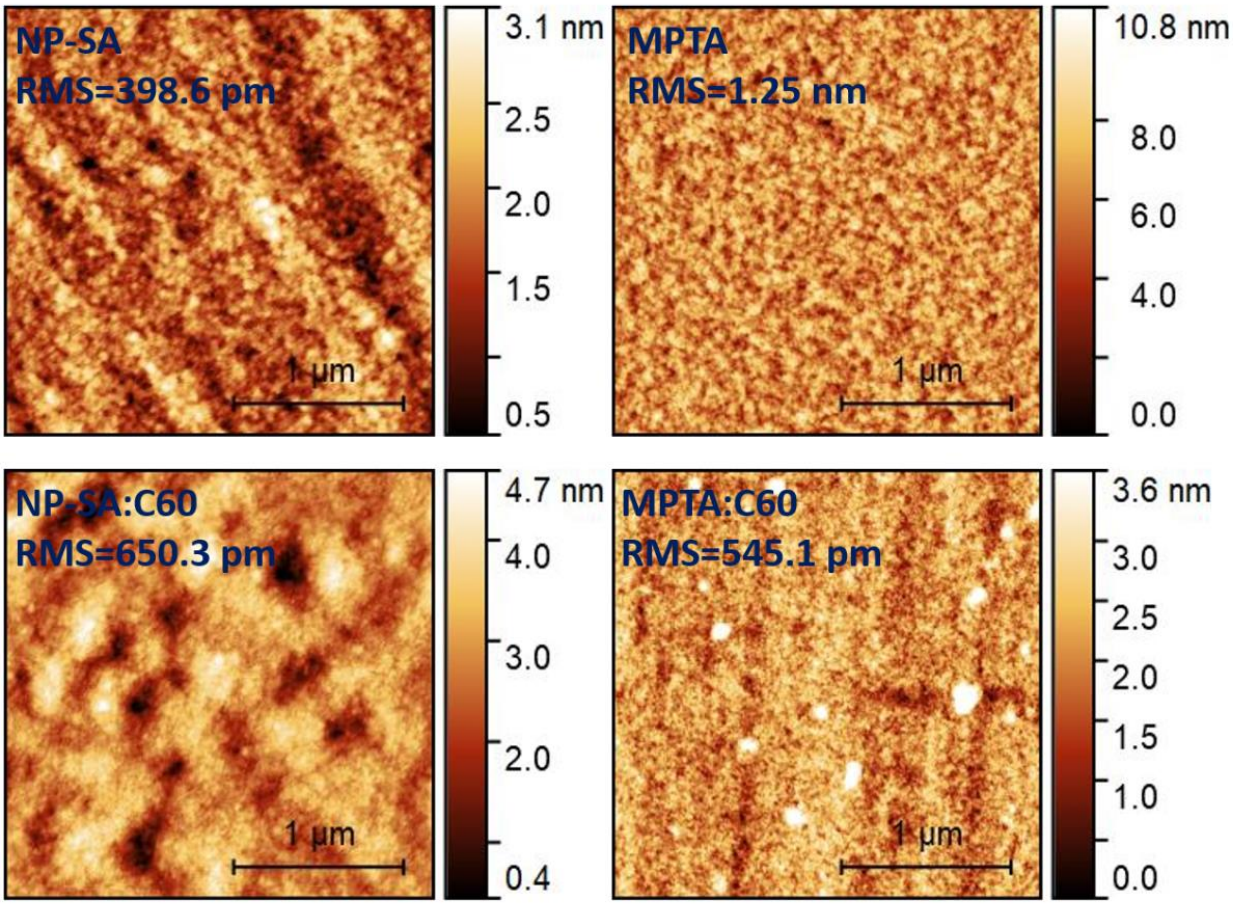

Supplementary Figure 12. AFM maps of neat donor films and respective blends with C_{60} on quartz substrate. Both donors show root mean square (RMS) roughness ≤ 1 nm in neat and blend films.

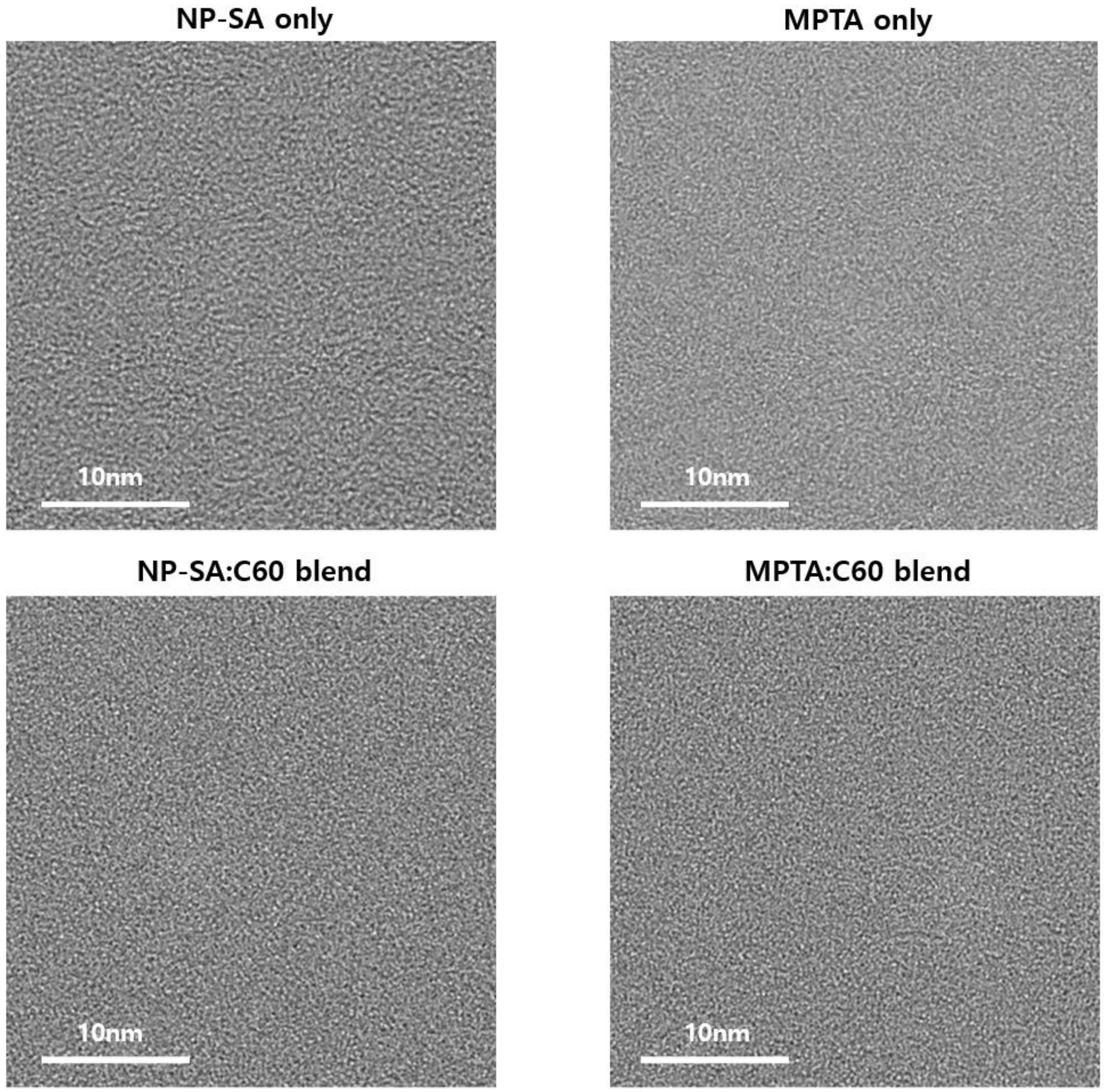

Supplementary Figure 13. TEM images of neat donor and their blend films, showing no measurable changes in blend morphology compared to neat films.

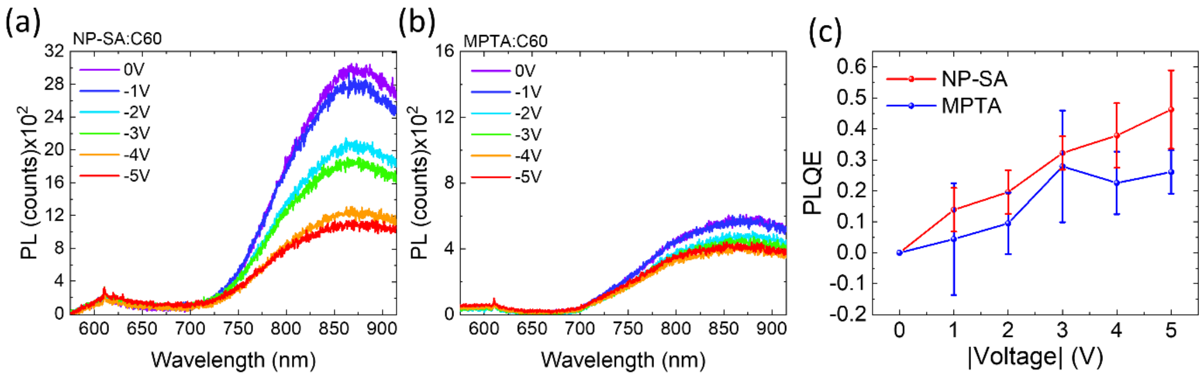

Supplementary Figure 14. (a)-(b) Bias-dependent PL for NP-SA:C_{60} and MPTA:C_{60} devices respectively, using 514 nm laser as excitation source and corrected for absorption. (c) PL quenching efficiency as a function of reverse bias applied to NP-SA:C_{60} and MPTA:C_{60} devices, averaged from 3-4 points on the devices, with error bars indicated as standard deviation. The PL quenching efficiency is calculated as PLQE = $\frac{PL_{V=0} - PL_V}{PL_{V=0}}$, with $PL_{V=0}$ and PL_V respectively being the PL at zero bias and at the applied reverse bias at the wavelength of maximum emission.

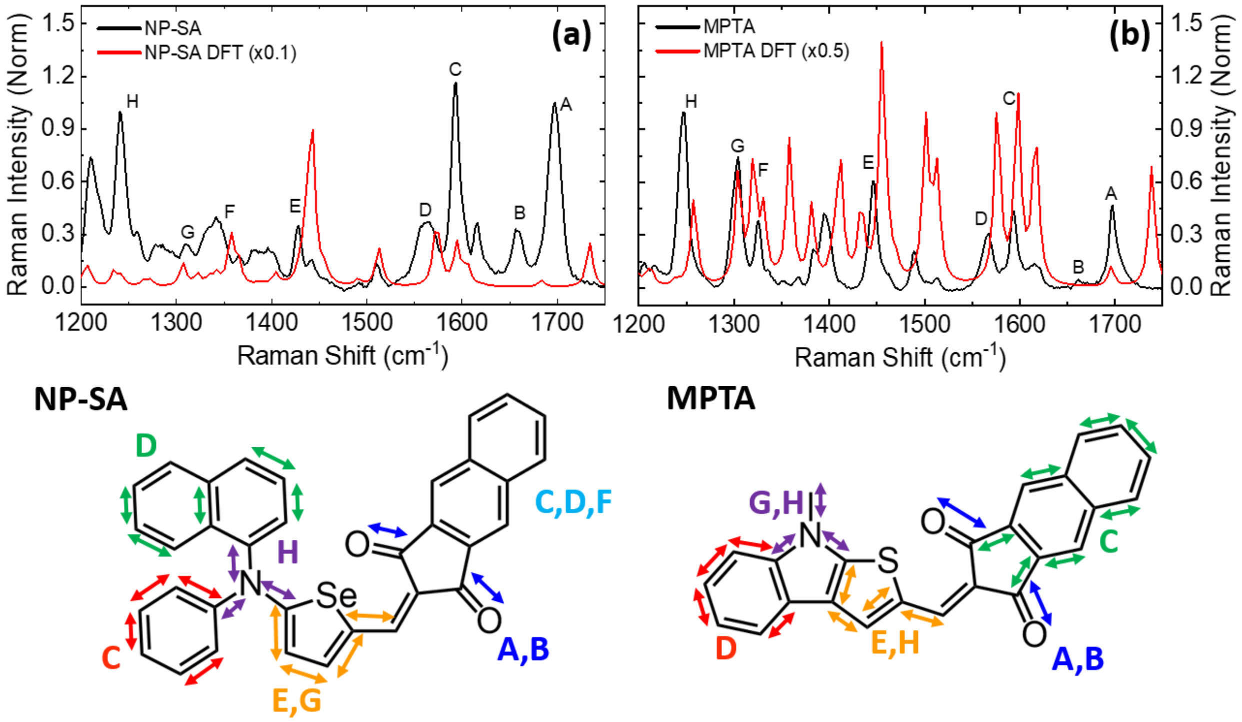

Supplementary Figure 15. Comparison of experimental and DFT simulated Raman spectra for NP-SA (a) and MPTA (b), with the peak assignment illustrated in the respective molecular structures.

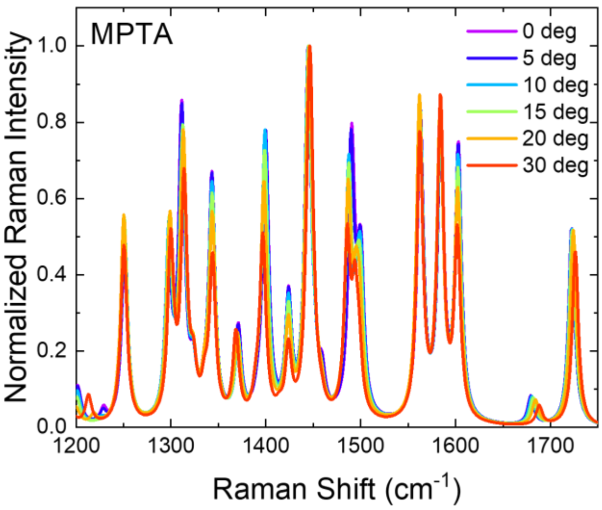

Supplementary Figure 16. DFT simulation of MPTA Raman spectra for different twisting angles between the donor and the acceptor unit.

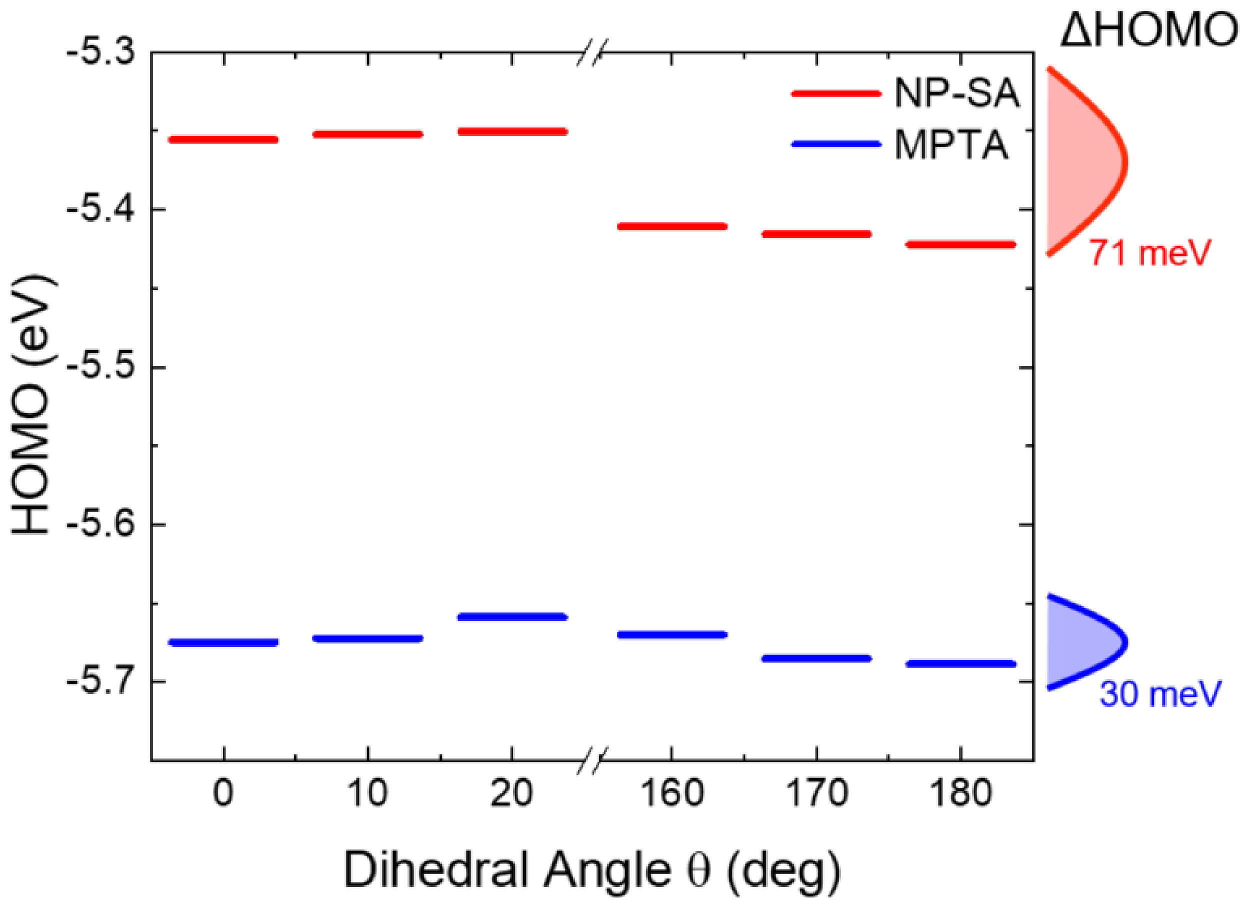

Supplementary Figure 17. DFT simulated HOMO levels for NP-SA and MPTA for the near-equilibrium configurations 20º around the possible isomeric forms. The HOMO dispersion is indicated on the right.

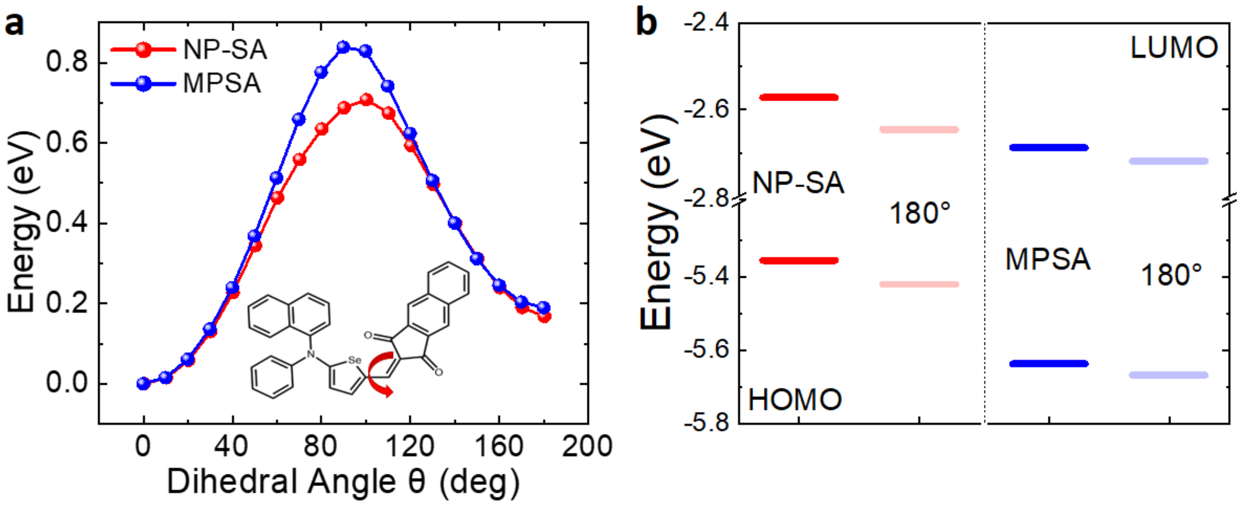

Supplementary Figure 18. DFT calculations for NP-SA compared with the selenium substituted version of MPTA. (a) Potential energy scan as a function of alkene bond dihedral angle between donor and acceptor units (as indicated in the inset) for NP-SA and the selenated analogous of MPTA (referred to as MPSA). (b) HOMO and LUMO energy levels for optimized geometries of NP-SA and MPSA and their isomers, respectively with 0 and 180º dihedral between donor and acceptor units.

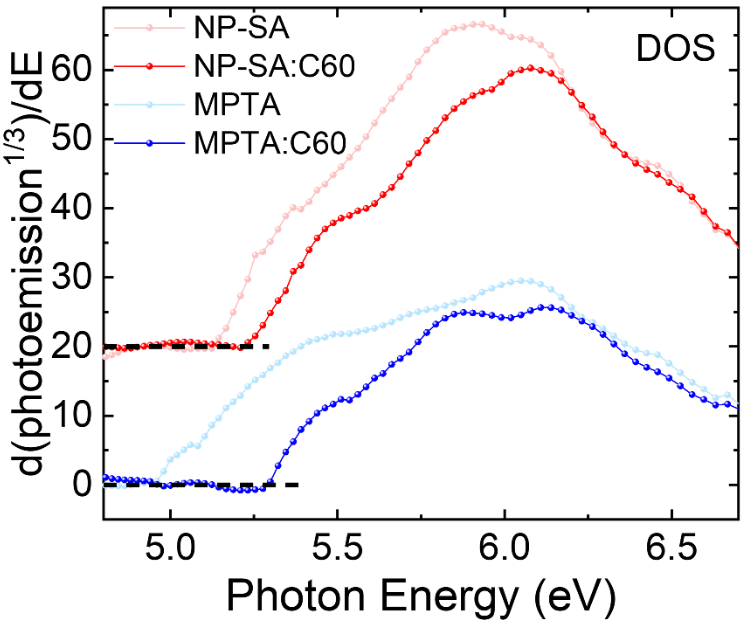

Supplementary Figure 19. Density of states from photoemission spectra of neat and blended NP-SA and MPTA, with respective baselines indicated as black dashed lines. The spectra have been separated for clearer display by vertical shift of NP-SA pair.

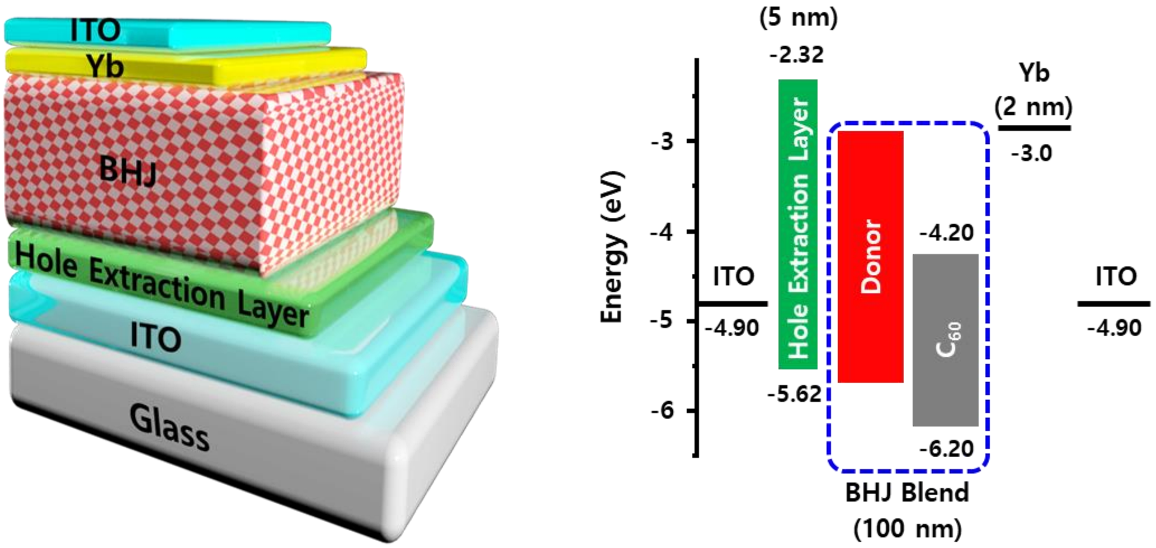

Supplementary Figure 20. Device structure of organic photodetector with an ITO-coated glass substrate, the hole extraction layer, the organic BHJ layer of Donor:C_{60}, electron extraction layer, Ytterbium (Yb) and an ITO electrode. The energetic diagram of device structure is shown on the right.

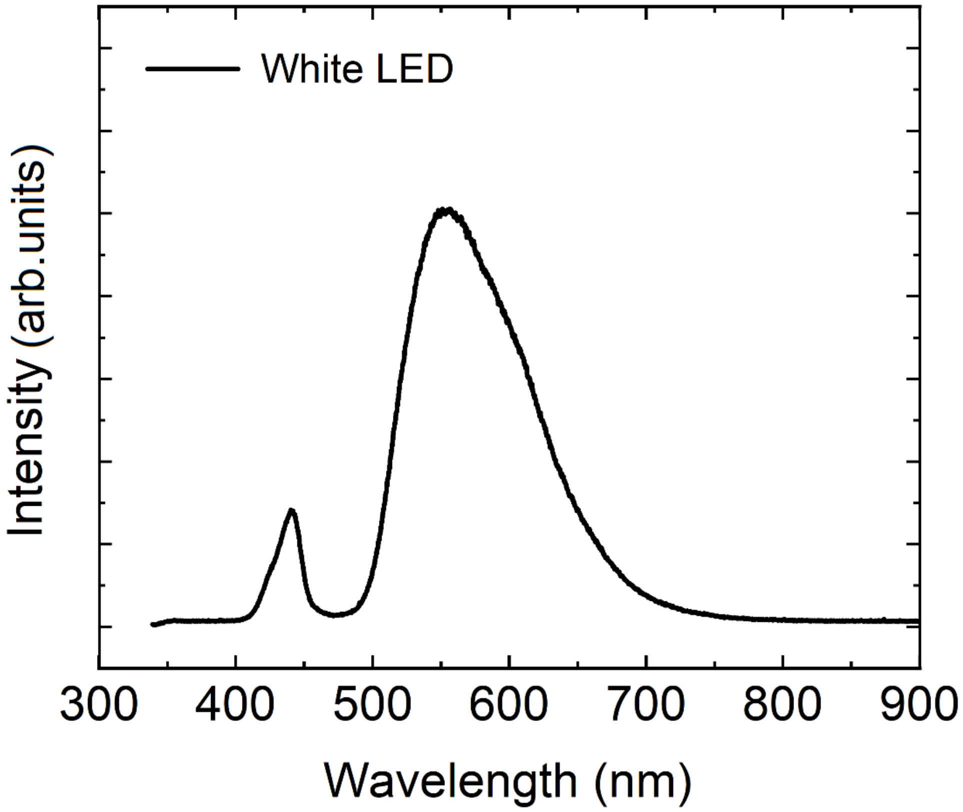

Supplementary Figure 21. Normalized intensity spectrum of the white LED used for transient optoelectronic measurements.

Supplementary Tables

**Peaks (±1 cm^{-1})**	**NP-SA – Vibrational Assignments**
H (1240)	C-N stretch; selenophene C=C stretch
**Core Group**	
G (1310)	Selenophene C=C; C-N and alkene stretch
F (1341)	H-wagging of selenophene and alkene stretch
E (1428)	Selenophene C-C intraunit; neighboring C-N and C-C interunit
**End Group**	
D (1563)	Naphthalene intraunit stretch; strong alkene stretch
C (1594)	Phenyl intraunit stretch and interunit C-N; C-C stretch in acceptor unit
B (1656)	C=O, antisymmetric stretch
A (1697)	C=O, symmetric stretch
**Peaks (±1 cm^{-1})**	**MPTA – Vibrational Assignments**
H (1246)	C-N stretch; selenophene C=C and neighboring C-C, C-N
**Core Group**	
G (1304)	C-N stretch; H-wagging of thiophene, donor unit and alkene
F (1325)	H-wagging of thiophene and alkene; donor unit C-C
E (1445)	Thiophene C=C; donor unit C=C and alkene stretch
**End Group**	
D (1567)	Donor unit phenyl stretch; alkene and acceptor unit C-C, C=C stretch
C (1594)	Acceptor unit C-C, C=C stretch; alkene and donor unit phenyl stretch
B (1662)	C=O, antisymmetric stretch
A (1697)	C=O, symmetric stretch

Supplementary Table 1. Detailed assignment of Raman vibrational modes of NP-SA and MPTA, referred to Figure S14.

Supplementary Notes

Supplementary Note 1. Assuming the charge carrier transport is via the hopping process and occurs only from a single trapped site to the mobility edge, thus the hopping probability (P) of trapped carrier scales with the hopping energy barrier (E_a), which means the energy difference between the mobility edge and the trap site.

$$P \propto \exp\left(\frac{-E_a}{kT}\right) \qquad (1)$$

Where k is Boltzmann's constant, T the temperature.

For a two states system with the energy level of E_1 and E_2 respectively, thus the hopping rate ratio of these two trapped carriers is $\frac{P_1}{P_2} \propto \exp\left(\frac{E_1 - E_2}{kT}\right)$. Therefore, the mobility, which is proportional to the hopping rate, and the transport time, which is reversely proportional to the mobility, can be approximated

$$\frac{\mu_1}{\mu_2} = \frac{t_2}{t_1} \propto \exp\left(\frac{E_{12}}{kT}\right) \qquad (2)$$

Given the transport time determined via the charge extraction (see Figure 2 and corresponding discussion in the main text), the energy difference between the two states can be estimated.

Supplementary Methods

Materials

All chemicals and reagents from commercial sources were used without further purification. All anhydrous solvents for the synthesis were purchased from Sigma-Aldrich. 2-((5-(naphthalen-1-yl(phenyl)amino)selenophen-2-yl)methylene)-1H-cyclopenta[b]naphthalene-1,3(2H)-dione, NP-SA was synthesized according to reference.[1,2] 2-((8-methyl-8H-thieno[2,3-b]indol-2-yl)methylene)-1H-cyclopenta[b]naphthalene-1,3(2H)-dione, MPTA was synthesized as shown in scheme below.[1-3]

Synthetic scheme

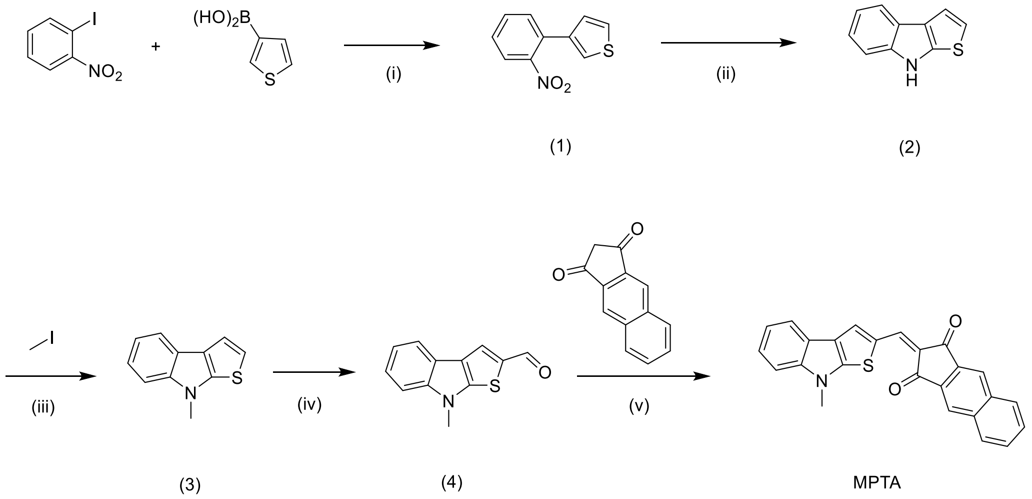

Supplementary Figure 22. Synthetic procedures of MPTA, (i) $Pd(PPh_3)_4$, K_2CO_3, DMF/H_2O, 90 °C, 12 h, (ii) PhMgBr, THF, 0 °C, (iii) KOH, DMSO, 30 °C, 5 h, (iv) $POCl_3$, DMF, DCM, (v) EtOH, 50 °C, 12 h.

(1) Synthesis of 3-(2-nitrophenyl)thiophene)

6.0 g (24.1 mmol) of 1-iodo-2-nitrobenzene, 4.0 g (31.3 mmol) of thiophene-3-yl boronic acid, 1.81 g (1.57 mmol) of tetrakis(triphenylphosphine)palladium (0) and 13.3 g (96.4 mmol) of K_2CO_3 are dissolved in 100 ml of DMF and 100 ml of water and then, reacted therewith at 90 °C for 12 hours. A product extracted with diethyl ether at room temperature is separated and purified through silica gel column chromatography (a volume ratio of ethyl acetate : hexane = 1:8) to obtain 4.36 g (Yield = 88.2%) of Compound (1), 3-(2-nitrophenyl)thiophene) as a yellow oil. ^{1}H NMR (500 MHz, CD_2Cl_2) δ = 7.81 (d, 1H, J = 8.5 Hz), 7.62 (t, 1H, J = 7.5 Hz), 7.54-7.48 (m, 2H), 7.45-7.43 (m, 1H), 7.39-7.37 (m, 1H), 7.10 (d, 1H, J = 5.0 Hz). ^{13}C NMR (500 MHz, CD_2Cl_2) δ = 137.2, 132.4,

131.8, 130.6, 128.2, 127.4, 127.3, 126.3, 123.8, 123.4 ppm; HRMS (ESI+): calculated for $C_{10}H_7NO_2S$ [M + H+], 205.02; measured, 206.06.

(2) Synthesis of 8H-thieno[2,3-b]indole

4.0 g (19.5 mmol) of 3-(2-nitrophenyl)thiophene) is dissolved in 250 ml of dry THF and cooled down to 0 °C, and 18.9 ml (58.5 mmol) of PhMgBr (1.0 M in a THF solution is slowly added thereto in a dropwise fashion. While added over 10 minutes as above, a temperature of the solution is internally controlled not to be over 3 °C. The solution is reacted at 0 °C for 5 minutes, and 50 ml of NH_4Cl saturated solution is added thereto. 500 ml of water is added thereto, and an organic layer therefrom is washed with an aqueous sodium chloride solution, three times extracted with ethyl acetate, and dried with anhydrous magnesium sulfate. A product therefrom is separated and purified through silica gel column chromatography (a volume ratio of ethyl acetate : hexane = 1:5) to obtain 2.88 g (Yield = 85.2%) of Compound (2), 8H-thieno[2,3-b]indole as a white solid. M.p. 192 °C; ^{1}H NMR (500 MHz, CD_2Cl_2) δ = 8.45 (br s, 1H), 7.82 (d, 1H, J = 6.5 Hz), 7.46 (d, 1H, J = 7.0 Hz), 7.38 (d, 1H, J = 6.0 Hz), 7.28-7.26 (m, 1H), 7.21-7.19 (m, 1H), 6.93 (d, 1H, J = 6.0 Hz). ^{13}C NMR (500 MHz, CD_2Cl_2) δ = 142.2, 141.1, 125.3, 122.3, 122.1, 119.9, 119.1, 117.7, 117.0, 111.2 ppm; HRMS (ESI+): calculated for $C_{10}H_7NS$ [M + H+], 173.03; measured, 173.98.

(3) Synthesis of 8-methyl-8H-thieno[2,3-b]indole

2.8 g (16.2 mmol) of 8H-thieno[2,3-b]indole and 9.07 g (161.6 mmol) of potassium hydroxide are dissolved in 70 ml of dimethyl sulfoxide, and 15.7 g (48.5 mmol) of iodomethane is added thereto in a dropwise fashion. The obtained mixture is stirred at 30 °C for 5 hours. 250 ml of water is added thereto, and dichloromethane is used for an extraction. An extract therefrom is dried with anhydrous magnesium sulfate, separated and purified through silica gel column chromatography (a volume ratio of hexane : dichloromethane = 5:1) to obtain 2.59 g (Yield = 85.6%) of Compound (3), 8-methyl-8H-thieno[2,3-b]indole as a white solid. M.p. 67 °C; ^{1}H NMR (500 MHz, CD_2Cl_2) δ = 7.82 (d, 1H, J = 8.0 Hz), 7.40 (t, 2H, J = 5.0 Hz), 7.33-7.30 (t, 1H), 7.21-7.19 (m, 1H), 6.93 (d, 1H, J = 5.0 Hz), 3.87 (s. 3H). ^{13}C NMR (500 MHz, CD_2Cl_2) δ = 144.7, 142.5, 122.8, 121.8, 119.1, 118.2, 118.0, 116.3, 116.0, 109.0, 32.7 ppm; HRMS (ESI+): calculated for $C_{11}H_9NS$ [M + H+], 187.05; measured, 187.93.

(4) Synthesis of 8-methyl-8H-thieno[2,3-b]indole-2-carbaldehyde

2.0 ml of phosphoryl chloride is added in a dropwise fashion to 10.0 ml of N,N-dimethylformamide at -15 °C and then, stirred at room temperature for 2 hours. This is slowly added in a dropwise fashion to a mixture of 100 ml of dichloromethane and 2.5 g of Compound (3) at -15 °C and then, stirred at room temperature for 30 minutes and concentrated under a reduced pressure. Then, 150 ml of water is added thereto, and an aqueous sodium hydroxide solution is added thereto until pH becomes 14 and then, stirred at room temperature (24 °C) for 2 hours. An organic layer extracted with dichloromethane is washed with an aqueous sodium chloride solution and then, dried with anhydrous magnesium sulfate. A product therefrom is separated and purified through silica gel column chromatography (a volume ratio of hexane : ethyl acetate = 4:1) to obtain 2.39 g (Yield = 83.3%) of Compound (4), 8-methyl-8H-selenopheno[2,3-b]indole-2-carbaldehyde as a green solid. M.p. 102 ℃; ^{1}H NMR (500MHz, CD_2Cl_2) δ = 9.86 (s, 1H), 8.09 (s, 1H), 7.88 (d, 1H, *J* = 7.5 Hz), 7.45-7.40 (m, 2H), 7.31-7.28 (m, 1H), 3.88 (s, 3H). ^{13}C NMR (500 MHz, CD_2Cl_2) δ = 182.9, 151.4, 143.4, 136.1, 129.8, 123.9, 123.7, 122.3, 120.9, 119.8, 109.8, 32.8 ppm; HRMS (ESI+): calculated for $C_{12}H_9NOS$ [M + H+], 215.04; measured, 215.80.

(5) Synthesis of 2-((8-methyl-8H-thieno[2,3-b]indol-2-yl)methylene)-1H-cyclopenta[b]naphthalene-1,3(2H)-dione, MPTA

2.3 g (10.68 mmol) of Compound (4) is suspended in ethanol, and 2.2 g (11.22 mmol) of 1H-cyclopenta[b]naphthalene-1,3(2H)-dione is added thereto and reacted therewith at 50 °C for 12 hours to obtain 3.46 g (Yield = 82.5％) of Compound (5), 2-((8-methyl-8H-thieno[2,3-b]indol-2-yl)methylene)-1H-cyclopenta[b]naphthalene-1,3(2H)-dione (MPTA) as a red solid. M.p. 315 ℃; ^{1}H-NMR (500 MHz, CD_2Cl_2): δ 8.37 (d, 2H, *J* = 12.5 Hz), 8.18 (s, 1H), 8.13-8.09 (m, 3H), 7.95 (d, 1H, *J* = 8.0 Hz), 7.70-7.68 (m, 2H), 7.46 (d, 2H, *J* = 4.0 Hz), 7.36-7.33 (m, 1H), 3.98 (s, 3H). ^{13}C NMR (600 MHz, CD_2Cl_2) δ = 144.3, 137.4, 136.3, 131.4, 130.2, 130.1, 128.7, 128.6, 124.9, 124.7, 122.7, 122.5, 121.6, 120.8, 117.5, 110.0, 32.7 ppm; HRMS (ESI+): calculated for $C_{25}H_{15}NO_2S$ [M + H+], 393.08; measured, 394.13. MALDI TOF MS: m/z 392.00. Elemental analysis: calculated for $C_{25}H_{15}NO_2S$: C - 76.32%, H - 3.84%, N - 3.56%, O - 8.13%; found: C - 76.07%, H - 3.83%, N - 3.58%, O - 8.12%.

Supplementary References

(1) Lee, G. H. *et al.* Green-light-selective organic photodiodes for full-color imaging. *Opt. Express* **27**, 25410 (2019)

(2) Kim, H. -J. *et al.* Harnessing intramolecular chalcogen-chalcogen bonding in merocyanines for utilizing in high-efficiency photon-to-current conversion optoelectronics. *Acs Appl. Mater. Interfaces* **14**, 4360 (2021)

(3) Gao. H. *et al.* Rapid synthesis of fused N-heterocycles by transition-metal-free electrophilic amination of arene C-H bonds. *Angew. Chem. Int. Ed.* **53**, 2701 (2014)
